# Gefährdet der Pflegepersonalmangel auf neonatologischen Intensivstationen die Versorgungssicherheit Neugeborener? Das Projekt „Maria und Josef“

**DOI:** 10.1007/s00103-023-03749-6

**Published:** 2023-08-10

**Authors:** Daniel Fichtner, Andreas W. Flemmer, Uli Fischer, Viola Koncz, Anna-Lisa Oechsle, Mathias Klemme

**Affiliations:** 1 Institut für Notfallmedizin und Medizinmanagement – INM, LMU Klinikum, Schillerstraße 53, 80336 München, Deutschland; 2 Neonatologie der Kinderklinik, Dr. von Haunersches Kinderspital & Perinatalzentrum, LMU Klinikum, München, Deutschland; 3 Stabsstelle klinische Pflegeforschung und Qualitätsmanagement, LMU Klinikum, München, Deutschland; 4 Stabsstelle Versorgungsmanagement Gesundheit und Pflege, Gesundheitsreferat Landeshauptstadt München, München, Deutschland; 5 Institut für soziale Pädiatrie und Jugendmedizin, Ludwig-Maximilians-Universität München, München, Deutschland

**Keywords:** Pflegepersonalmangel, Neonatologie, Intensivverlegung, Pflegenotstand, Intensivversorgung, Nursing shortage, Neonatology, Intensive patient transport, Nursing crisis, Intensive care

## Abstract

**Hintergrund:**

In Deutschland ist seit Jahren ein Pflegepersonalmangel zu beobachten, der insbesondere neonatologische Intensivstationen betrifft. Dabei wird vermutet, dass dieser Mangel zu reduzierten Versorgungskapazitäten führt, woraus Konsequenzen für die Versorgung von Neugeborenen resultieren. Diese werden mit einer retrospektiven Beobachtungsstudie am Beispiel der 3 neonatologischen Intensivstationen des LMU Klinikums München betrachtet.

**Methoden:**

Für den 4‑jährigen Beobachtungszeitraum 08/2017–05/2021 wurden Zeitreihendaten der „Qualitätssicherungs-Richtlinie Früh- und Reifgeborene“ (QFR-RL) des Gemeinsamen Bundesausschusses, Bettenauswertungen, Personalplanwerte, Abmeldedaten und rettungsdienstliche Einsatzdaten der Stationen herangezogen und mittels einer deskriptiven Statistik sowie einer 2‑stufigen Regressionsanalyse untersucht.

**Ergebnisse:**

Im Beobachtungszeitraum waren rund 21 % der notwendigen Pflegepersonalstellen unbesetzt, wobei die Qualitätsanforderungen gemäß QFR-RL weitgehend erfüllt waren. Um in Anbetracht des Personalmangels diese Anforderungen zu erfüllen, mussten jedoch verfügbare Betten gesperrt werden. Im Beobachtungszeitraum konnte sowohl eine Zunahme der Abmeldestunden der Stationen von der Bevölkerungsversorgung als auch ein Anstieg des relativen Risikos für eine Neugeborenen-Intensivverlegung beobachtet werden, wodurch im Mittel alle 3 Tage eine Verlegung resultierte.

**Diskussion:**

Die Anforderungen an die pflegerische Versorgungsqualität von Neugeborenen sind gesetzlich vorgeschrieben, sodass sich der Pflegepersonalmangel überproportional in einer Reduktion der Versorgungskapazität niederschlägt. So müssen Konsequenzen für die Versorgungssicherheit der Bevölkerung durch Klinikabmeldungen und ein Verlegungsrisiko regelhaft in Kauf genommen werden.

**Zusatzmaterial online:**

Zusätzliche Informationen sind in der Online-Version dieses Artikels (10.1007/s00103-023-03749-6) enthalten.

## Abstract (en)

**Background:**

Germany has been experiencing a dramatic shortage of nursing staff for years that particularly affects neonatal intensive care units (NICUs). It is assumed that this situation leads to reductions in bed capacities, resulting in negative effects on the healthcare of newborns. These were investigated through a retrospective observational study using the example of three NICUs at the University Hospital of Munich (LMU).

**Methods:**

For the four-year observation period from August 2017 to May 2021, time series data from the “Quality Assurance Guideline for Premature and Mature Infants” (QFR-RL) of the Federal Joint Committee, bed resource analysis, planned personnel statistics, clinical logout data, and rescue service data were mutually examined using descriptive statistics and regression analysis.

**Results:**

During the observation period, around 21% of the necessary nursing staff positions were vacant, although the quality of nursing care for newborns seemed to have been guaranteed. However, to ensure quality, given the staff shortage, several available beds had to be blocked. In this context, both an increase in the number of hours the wards were logged off from population care and an increase in the relative risk of neonatal intensive care transfer were observed, resulting in a transfer every three days on average.

**Discussion:**

A shortage of nursing staff reduces the neonatal hospital bed capacity, since neonatal nursing care quality is regulated by strict legally binding guidelines, the QFR-RL. This is why the consequences for the security of care for the population through hospital cancellations and a risk of transfer must be accepted on a regular basis.

## Hintergrund

In Deutschland besteht über viele Jahre ein zunehmender Pflegepersonalmangel im Gesundheitswesen, wobei hochspezialisierte Bereiche der Kliniken, wie die neonatologischen Intensivstationen als Spezialbereich der Kinder- und Jugendmedizin, besonders betroffen sind [1, 2]. Verschärft wurde dieser Pflegepersonalmangel in der Neonatologie nicht zuletzt durch die COVID-19-Pandemie sowie die saisonalen Viruswellen des respiratorischen Synzytial-Virus (RSV) [3, 4].

Die Auswirkungen des Pflegepersonalmangels werden bereits in verschiedenen wissenschaftlichen Arbeiten diskutiert. Entsprechend werden neben der Assoziation mit einer gesteigerten Patientenmortalität bei Erwachsenen [5, 6] auch Versorgungsengpässe für die Bevölkerung durch nicht betreibbare Betten aufgrund von Personalknappheit konstatiert [2, 7–9]. Diese Engpässe werden zudem häufig mit einem erhöhten Aufkommen an Verlegungen durch den öffentlichen Rettungsdienst und durch die mangelnde Versorgungskapazität in Zusammenhang gebracht [10–12]. Besonders kritisch sind Verlegungen von kranken Früh- und Neugeborenen zu bewerten, da sie mit einem signifikant erhöhten Risiko für das Kind einhergehen. Daher sind diese unmittelbaren postnatalen Intensivverlegungen zu vermeiden [10, 13–15]. Eine Verbesserung der Personalsituation hätte durch die daraus folgende Zunahme der betreibbaren Betten auf neonatologischen Intensivstationen das Potenzial, die Zahl an Neugeborenen-Intensivverlegungen zu senken.

Im Jahr 2021 wurden an den beiden Level-I-Perinatalzentren des LMU Klinikums insgesamt 4006 Kinder geboren [16]. Neben der Versorgung von Hochrisikogeburten ist das LMU Klinikum ein überregionaler Maximalversorger für die Versorgung schwerstkranker Mütter und Neugeborener aus der Region. Kranke Neugeborene werden auf insgesamt 3 neonatologischen Intensivstationen versorgt, welche mit einem gravierenden Pflegepersonaldefizit konfrontiert sind.

Pflegepersonaldefizite führen seit Jahren bereits deutschlandweit in zunehmenden Maß zu einer potenziell lebensbedrohlichen Reduktion der betreibbaren Betten [17]. In Anbetracht dieser Situation stehen allerdings bisher kaum flächendeckende und standardisiert erhobene Daten für Untersuchungen des Pflegepersonalmangels und der damit verbundenen Auswirkungen zur Verfügung [18]. Ziel des Forschungsprojekts „Maria und Josef“ war es, den Mangel an Pflegepersonal am Beispiel der neonatologischen Intensivstationen des LMU Klinikums anhand verschiedener standardisierter Datenquellen zu quantifizieren und im Weiteren Rückschlüsse auf die Versorgung der Bevölkerung zu ziehen.

## Methoden

### Studiensetting

Es wurde eine longitudinale retrospektive Beobachtungsstudie durchgeführt. Hierfür wurden für den Beobachtungszeitraum vom 01.08.2017 bis 31.05.2021 Zeitreihendaten auf Tagesebene für die 3 neonatologischen Intensivstationen des LMU Klinikums vollständig erhoben, wodurch 1400 Beobachtungstage resultierten. Diese Stationen befanden sich am Perinatalzentrum Campus Großhadern (*Station I10B*), im Dr. von Haunerschen Kinderspital (*Neonatologische Intensivpflegestation – NIPS*) und an der LMU Frauenklinik des Perinatalzentrums Campus Innenstadt (*Maistraße Intensivpflegestation – MIPS*) [19]. Für alle 3 Stationen resultieren somit insgesamt 4200 Beobachtungen.

### Datengrundlage

Als Datenquellen dienten die pflegerischen Dokumentationsdaten zur „Qualitätssicherungs-Richtlinie Früh- und Reifgeborene“ (QFR-RL; [20]), Bettenauswertungen, Pflegepersonalplanwerte des LMU Klinikums, rettungsdienstliche Einsatzdaten sowie Abmeldedaten des Leitstellen-Meldeportals „IVENA eHealth®“ [21], das die interdisziplinäre Aufnahmefähigkeit der Kliniken erfasst.

Bei der *QFR-RL* handelt es sich um eine Richtlinie des Gemeinsamen Bundesausschusses (G-BA), die unter anderem die pflegerische Versorgungsqualität der Neugeborenen durch Personalvorgaben für die Perinatalzentren sichern soll. Die Stationen sind dazu verpflichtet, die Anzahl der zu versorgenden Neugeborenen nach Versorgungsaufwand und den realen Personalbestand schichtgenau zu dokumentieren. Dem Versorgungsaufwand eines jeden Neugeborenen wird der notwendige Pflegepersonalschlüssel gemäß QFR-RL zugrunde gelegt, wobei die Richtlinie den Pflegenden keinen Spielraum lässt, den tatsächlichen Pflegeaufwand selbst einzuschätzen. Daraus resultiert eine verpflichtende Pflegepersonalanforderung pro Schicht, die sich durch die Anzahl der zu versorgenden Neugeborenen und deren Versorgungsanforderungen ergibt. Über das Verhältnis der tatsächlich eingesetzten Pflegekräfte zu den zur Versorgung gemäß QFR-RL notwendigen Pflegekräften kann eine *reale Personalerfüllungsquote (rPQ)* je Schicht betrachtet werden, welche für die deskriptive Analyse über die 3 Schichten pro Tag gemittelt wurde. (Im Gegensatz zur Berechnung der rPQ, bei der alle Neugeborenen der Station unabhängig vom Geburtsgewicht einbezogen werden, berücksichtigt die sog. Schichterfüllungsquote gemäß QFR-RL lediglich den Anteil der Schichten hinsichtlich der pflegerischen Versorgung von Frühgeborenen mit einem Geburtsgewicht unter 1500 g [20])*.*

Die *Bettenauswertung* des LMU Klinikums umfasst Daten zur Anzahl der aufstellbaren Betten, der gesperrten Betten und deren Bettensperrgründe pro Tag. Die Anzahl betreibbarer Betten ergibt sich durch die Anzahl der aufstellbaren Betten abzüglich der gesperrten Betten. Der Parameter *Bettenauslastung* berechnet sich aus dem Verhältnis der Anzahl versorgter Neugeborener pro Tag und der Anzahl betreibbarer Betten pro Tag.

Die *Pflegepersonalplanwerte* enthalten Daten zu den notwendigen Vollkosten-Pflegepersonalstellen (VK-Stellen) und zu den besetzten VK-Stellen pro Monat je Station. Die notwendigen VK-Stellen orientieren sich dabei an der Anzahl an Pflegepersonal, das gemäß Pflegepersonalkalkulation prospektiv notwendig ist, um die physisch aufstellbaren Betten betreuen zu können. Pro aufstellbarem Bett wird hierbei ein durchschnittlicher Pflegepersonalschlüssel von 1:2 für die Personalplanung angenommen. Da es sich um eine prospektive Planung handelt, kann der tatsächliche Pflegepersonalbedarf hiervon abweichen. Die errechnete Quote aus planerischem Personalbestand und -bedarf ergibt die *planerische Personalerfüllungsquote (pPQ)*.

Die *rettungsdienstlichen Einsatzdaten* der Integrierten Leitstelle München enthalten alle durchgeführten Neugeborenen-Intensivverlegungen mit Ausgangsort einer der neonatologischen Intensivstationen des LMU Klinikums. Daraus wurde ein *Verlegungsaufkommen* pro Tag je Station errechnet.

Das Leitstellen-Meldeportal *IVENA eHealth®*, das dazu dient, die Aufnahmefähigkeit der Kliniken zu erfassen und dadurch die rettungsdienstlichen Patientenströme in der Integrierten Leitstelle zu steuern, enthält Daten zu den abgemeldeten Versorgungsstunden der 3 neonatologischen Intensivstationen, d. h., dass die Stationen in dieser Zeit nicht für die Bevölkerungsversorgung zur Verfügung standen. Für jede Station werden die Abmeldestunden pro Tag erfasst. Diese wurden zu maximal möglichen 24 Versorgungsstunden pro Tag ins Verhältnis gesetzt, wodurch der *Anteil der abgemeldeten Versorgungsstunden *pro Tag im Verlauf beurteilt werden konnte.

### Studienhypothese

In dieser Untersuchung soll folgende Hypothese geprüft werden: Ein Rückgang der pPQ führt nahezu ausschließlich zu einer Einschränkung der Bettenkapazität, da die Versorgungsqualität der Neugeborenen im Sinne des durch die QFR-RL vorgegebenen Pflegepersonalschlüssels nicht verändert werden darf. Dies führt zu einem Anstieg der Bettenauslastung auf neonatologischen Intensivstationen. Hieraus resultieren vermehrt Abmeldestunden der Stationen von der Versorgung der Bevölkerung sowie eine höhere Anzahl an Neugeborenen-Intensivverlegungen aus Kapazitätsgründen.

### Statistik

Zur Überprüfung der Studienhypothese wurde neben einer deskriptiven Statistik auch eine 2‑stufige Regressionsanalyse durchgeführt. Zum einen wurde die Bettenauslastung als Zielvariable durch die pPQ und die Anzahl versorgter Neugeborener modelliert. Zum anderen wurde der Effekt der Bettenauslastung auf das Verlegungsaufkommen durch eine Negativ-Binomialregression untersucht. Da die Beobachtungstage von Zeitreihendaten grundsätzlich nicht als statistisch unabhängig voneinander betrachtet werden können, ist von einer zeitlichen Abhängigkeit bzw. von einer Autokorrelation der Beobachtungstage auszugehen [22]. Diese unterstellte zeitliche Abhängigkeit wurde bei der Modellierung der Bettenauslastung als Zielvariable mittels Durbin-Watson-Tests überprüft [22] und das Modell mittels der Methodik der Cochrane-Orcutt-Transformation [23] um den zeitlichen Effekt adjustiert. In der Modellierung des Verlegungsaufkommens als Zielvariable durch die Negativ-Binomialregression wurde hingegen die Autokorrelation durch die Aufnahme dieser zeitlichen Effekte im Regressionsmodell überprüft. Entsprechend wurde sowohl der Effekt des absoluten Werts der vorherigen Zeiteinheit an sich („past_obs“) als auch des bedingten Erwartungswerts der vorherigen Zeiteinheit („past_mean“) auf das Verlegungsaufkommen als Kovariablen in das Modell aufgenommen. Um zudem einen möglichen negativen Effekt auf das Verlegungsaufkommen korrekt zu modellieren, ohne dass ein negatives Verlegungsaufkommen resultiert, und daher das Modell besser zu spezifizieren, wurde die Linkfunktion logarithmiert. Dadurch entsteht ein Faktor, mit dem das Verlegungsaufkommen multipliziert und als ein relatives Risiko für eine Verlegung interpretiert werden kann [24]. Da die Verlegungen oder die Verlegungsentscheidungen in der Frühschicht getroffen wurden, wurden für diese Modellierung nicht die Tagesmittelwerte aus der QFR-RL-Dokumentation, sondern die entsprechenden Werte der Frühschicht berücksichtigt.

## Ergebnisse

### Deskriptive Statistik

**Figure 1:**
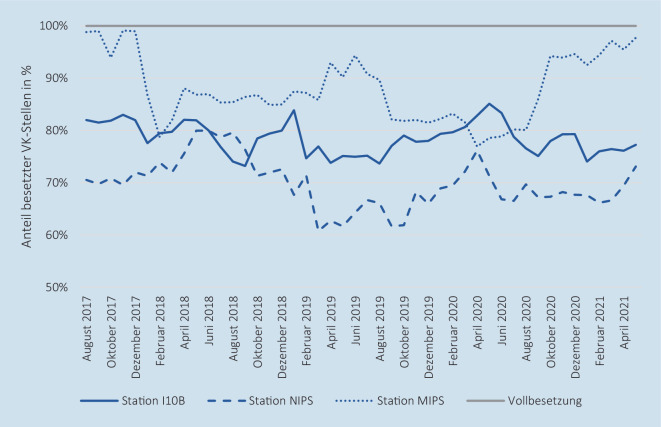

In der Tab. 1 werden die wesentlichen Lage- und Streuparameter der betrachteten Variablen aufgeführt. Bei Betrachtung aller 3 Stationen waren durchschnittlich 88,2 VK-Stellen von im Mittel 112,25 benötigten VK-Stellen besetzt, wodurch eine mittlere planerische Personalerfüllungsquote (pPQ) von 78,57 % (Minimum: 73,84 %, Maximum: 83,80 %) resultiert. Somit waren im Mittel 21 % der VK-Stellen (24,05 VK-Stellen) unbesetzt, wobei nur die *Station MIPS* mit einer maximalen pPQ von 99,13 % am Ende des Zeitraums die pflegerische Vollbesetzung tangierte. Die *Station NIPS* war mit einer mittleren pPQ von 69,76 % personell am schlechtesten besetzt. In Abb. 1 ist die zeitliche Entwicklung der pPQ als Monatsmittel dargestellt.

**Table 1:** 

Variable (alle Standorte) *Station I10B* *Station NIPS* *Station MIPS*	Erläuterung	Mittelwert	SD	Median	Min.	Max.
**Verlegungen** *Station I10B* *Station NIPS* *Station MIPS*	Anzahl durchgeführter Neugeborenen-Intensivverlegungen	**0,30** 0,16 0,03 0,11	**0,61** 0,44 0,18 0,36	**0** 0 0 0	**0** 0 0 0	**4** 3 2 3
**Anzahl versorgter Neugeborener** *Station I10B* *Station NIPS* *Station MIPS*	Anzahl pro Tag versorgter Neugeborener	**22,05** 8,21 6,58 7,27	**2,81** 1,41 1,11 1,47	**22,00** 8,00 6,67 7,33	**13,00** 4,67 1,33 2,33	**29,33** 13,00 9,33 12,00
**Anzahl aufstellbare Betten** *Station I10B* *Station NIPS* *Station MIPS*	Anzahl physisch vorhandener aufstellbarer Intensivbetten	**39,11** 15,89 10,22 13,00	**0,31** 0,31 0,62 0,00	**39,00** 16,00 10,00 13,00	**39,00** 15,00 10,00 13,00	**40,00** 16,00 12,00 13,00
**Anzahl gesperrte Betten aufgrund Pflegepersonalmangels**^a^ *Station I10B* *Station NIPS* *Station MIPS*	Anzahl gesperrter Intensivbetten ausschließlich aufgrund des Mangels an Pflegepersonal	**13,72** 6,62 2,90 4,19	**3,11** 1,44 0,98 1,41	**14,00** 7,00 3,00 5,00	**5,00** 3,00 0,00 0,00	**20,00** 10,00 5,00 8,00
**Anteil betreibbarer Betten** *Station I10B* *Station NIPS* *Station MIPS*	Quotient der betreibbaren und aufstellbaren Betten	**64,95** **%** 58,24 % 71,76 % 67,75 %	**7,80** **%** 9,45 % 8,70 % 10,87 %	**64,10** **%** 56,25 % 70,00 % 61,54 %	**48,72** **%** 37,50 % 58,33 % 29,17 %	**87,18** **%** 81,25 % 100,00 % 100,00 %
**Bettenauslastung** *Station I10B* *Station NIPS* *Station MIPS*	Anteil betreibbarer Betten, der zur Versorgung belegt ist	**87,28** **%** 89,55 % 90,78 % 83,36 %	**9,75** **%** 13,42 % 16,23 % 15,55 %	**87,65** **%** 88,89 % 94,44 % 83,33 %	**56,79** **%** 45,45 % 19,05 % 29,17 %	**116,67** **%** 150,00 % 150,00 % 128,57 %
**Anzahl notwendiger Pflegepersonalstellen** *Station I10B* *Station NIPS* *Station MIPS*	Anzahl prospektiv notwendiger VK-Stellen zum Betrieb der aufstellbaren Betten	**112,25** 49,29 32,52 30,44	**1,80** 0,00 0,53 1,78	**111,49** 49,29 32,92 29,28	**111,49** 49,29 31,83 29,28	**117,39** 49,29 32,92 35,18
**Anzahl besetzter Pflegepersonalstellen** *Station I10B* *Station NIPS* *Station MIPS*	Anzahl besetzter VK-Stellen	**88,20** 38,70 22,67 26,83	**3,53** 1,50 1,33 3,07	**88,06** 38,76 22,47 26,42	**82,33** 36,08 20,00 22,53	**96,53** 41,95 25,45 34,38
**pPQ** *Station I10B* *Station NIPS* *Station MIPS*	Anteil der besetzten an den notwendigen VK-Stellen	**78,57** **%** 78,51 % 69,76 % 87,98 %	**2,63** **%** 3,04 % 4,75 % 6,35 %	**78,72** **%** 88,89 % 69,54 % 86,78 %	**73,84** **%** 45,45 % 60,75 % 76,93 %	**83,80** **%** 85,11 % 79,96 % 99,13 %
**Planerisches Personaldefizit** *Station I10B* *Station NIPS* *Station MIPS*	Anzahl besetzter VK-Stellen abzüglich der Anzahl benötigter VK-Stellen	**−24,05** −10,59 −9,85 −3,61	**2,88** 1,50 1,65 1,85	**−24,11** −10,53 −10,01 −4,06	**−29,16** −13,21 −12,92 −6,76	**−18,11** −7,34 −6,38 −0,27
**rPQ** *Station I10B* *Station NIPS* *Station MIPS*	Anteil der zur Versorgung eingesetzten an den benötigten Pflegekräften	**91,83** **%** 96,05 % 90,16 % 94,41 %	**11,23** **%** 16,89 % 20,62 % 21,37 %	**91,53** **%** 94,28 % 86,96 % 90,16 %	**64,86** **%** 52,63 % 50,00 % 52,94 %	**158,97** **%** 187,50 % 257,14 % 222,22 %
**Reales Personaldefizit** *Station I10B* *Station NIPS* *Station MIPS*	Anzahl eingesetzter abzüglich der zur Versorgung benötigten Pflegekräfte	**−1,25** −0,35 −0,52 −0,39	**1,57** 0,90 0,79 0,76	**−1,17** −0,33 −0,50 −0,50	**−6,50** −4,50 −3,17 −2,83	**3,83** 2,33 2,00 2,00
**Anteil abgemeldete Versorgungsstunden** *Station I10B* *Station NIPS* *Station MIPS*	Anteil der Abmeldestunden an den maximalen Versorgungsstunden je Station	**36,45** **%** 29,84 % 54,76 % 24,75 %	**26,99** **%** 37,00 % 41,42 % 35,26 %	**33,33** **%** 0,00 % 64,24 % 0,00 %	**0,00** **%** 0,00 % 0,00 % 0,00 %	**100,00** **%** 100,00 % 100,00 % 100,00 %

*N* = 1400 Beobachtungstage, *I10B* Neonatologische Intensivstation (LMU Klinikum - Campus Großhadern), *MIPS* Maistraße Intensivpflegestation (LMU Frauenklinik), *NIPS* Neonatologische Intensivpflegestation (Dr. von Haunersches Kinderspital), *pPQ* planerische Personalerfüllungsquote, *rPQ* reale Personalerfüllungsquote, *SD* Standardabweichung, *VK-Stellen* Vollkosten-Pflegepersonalstellen

^a^ Weitere in dieser Variable nicht betrachtete Bettensperrgründe sind: Isolation, medizinische Gründe, medizinisch-soziale Gründe, bauliche Gründe und sonstige Gründe

**Figure 2:**
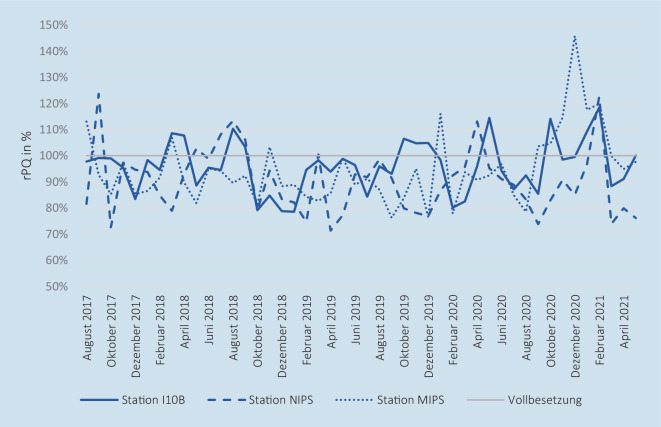

Bei Betrachtung der pflegerischen Versorgungsqualität in Form der realen Personalerfüllungsquote (rPQ) zeigte sich mit einem Mittelwert aller Standorte von 90,63 % ein deutlich höherer Durchschnitt als bei der pPQ (78,57 %). Im Maximum wurden auch Tage mit einer rPQ über 100 % (158,97 %) beobachtet. Ungefähr 75 % der Beobachtungstage wiesen eine pflegerische Unterbesetzung im Sinne der rPQ im Tagesmittel auf. Die Situation im zeitlichen Verlauf ist je Station in Abb. 2 veranschaulicht.

**Figure 3:**
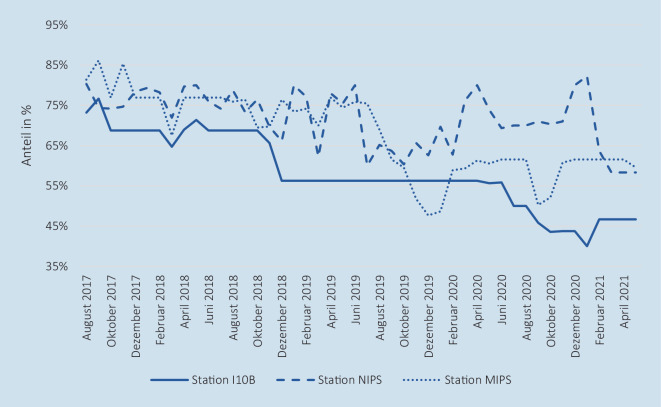

Auf allen 3 Stationen zusammen waren durchschnittlich 25,39 neonatologische Intensivbetten (Minimum: 19, Maximum: 34) von 39,11 im Mittel maximal aufstellbaren Betten betreibbar. Dies entspricht einem Anteil von rund 65 %. Zudem war festzustellen, dass der Anteil betreibbarer Betten im Verlauf des Beobachtungszeitraums deutlich gesunken ist, wobei dieser im Minimum bei Betrachtung aller Standorte bei 48,72 % im Monatsmittel lag. In Abb. 3 ist der zeitliche Verlauf des Anteils betreibbarer Betten dargestellt. Auf der *Station I10B* war dieser Anteil mit durchschnittlich 58,24 % am geringsten. Bei Betrachtung der Bettensperrgründe stellte sich zudem heraus, dass der Großteil der Sperrungen (98,91 %) auf den Pflegepersonalmangel zurückzuführen war. Der Verlauf der Anzahl gesperrter Betten aufgrund von Pflegepersonalmangel ist im Onlinematerial in *Zusatzabbildung 8 *und der zeitliche Verlauf sowohl der Anzahl aufstellbarer Betten als auch der Anzahl betreibbarer Betten je Station ist in *Zusatzabbildung 7* dargestellt.

An allen 3 Standorten zusammen wurden im Mittel rund 22 Neugeborene und im Maximum rund 29 Neugeborene pro Tag versorgt. Im Minimum wurden 13 Neugeborene versorgt. Der Verlauf der Anzahl versorgter Neugeborener je Station als Monatsmittel ist in *Zusatzabbildung 3* und die Entwicklung der Bettenauslastung im Verlauf in *Zusatzabbildung 6 *dargestellt. Dabei ist zu erkennen, dass die Bettenauslastung an allen 3 Standorten bei durchschnittlich 87,28 % (Minimum: 56,79 %, Maximum: 116,67 %) lag, wobei auf allen 3 Stationen gemäß Tab. 1 zeitweise auch eine deutliche Überbelegung zu beobachten war.

**Figure 4:**
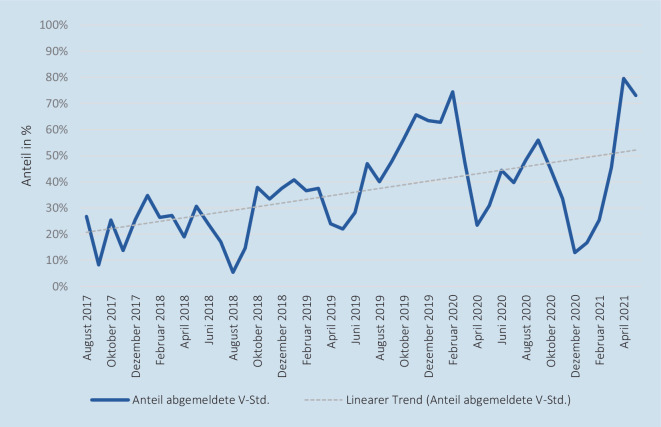

Bei Betrachtung der abgemeldeten Versorgungsstunden aller 3 Stationen wurden im Beobachtungszeitraum im Mittel 36,45 % der Versorgungsstunden abgemeldet, wobei maximal 72 Versorgungstunden bei 3 Stationen mit jeweils 24 Versorgungsstunden pro Tag zur Verfügung standen. Den höchsten durchschnittlichen Abmeldeanteil wies die *Station NIPS* mit 54,76 % auf, während auf der *Station I10B* im Mittel 29,84 % und auf der *Station MIPS* 24,75 % der Versorgungsstunden pro Tag abgemeldet wurden. Im Maximum wurden bei Betrachtung aller 3 Standorte im Beobachtungszeitraum an einigen Tagen 100 % der Versorgungstunden abgemeldet, was einer vollständigen gleichzeitigen Abmeldung der 3 Stationen für die Versorgung der Bevölkerung entspricht. Im Zeitverlauf ist zudem der Trend zu einer Zunahme der Abmeldestunden erkennbar, welcher in Abb. 4 dargestellt ist und sich auf einen mittleren Anteil der abgemeldeten Versorgungsstunden von 50 % einzupendeln scheint.

**Figure 5:**
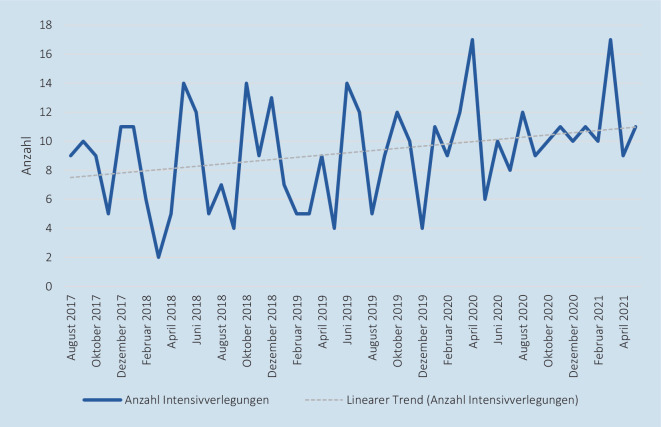

In den 1400 betrachteten Tagen kam es an den 3 Standorten zu insgesamt 425 Neugeborenen-Intensivverlegungen. Davon war der Großteil mit 52 % (221 Verlegungen) auf Verlegungen aus der *Station I10B* und mit 38 % (160 Verlegungen) aus der *Station MIPS* zurückzuführen. Die *Station NIPS* machte mit 44 Verlegungen einen deutlich niedrigeren Anteil aus. Bei der Gesamtbetrachtung der neonatologischen Intensivstationen des LMU Klinikums kam es im Mittel rund alle 3 Tage zu einer Neugeborenen-Intensivverlegung. Rund 2 Drittel dieser Verlegungen (65,88 % bzw. 280 Verlegungen) waren im Beobachtungszeitraum hinsichtlich der Quell- und Zielortrelation auf Verlegungen innerhalb der 3 neonatologischen Intensivstationen des LMU Klinikums zurückzuführen. Entsprechend wurden 34,12 % (145 Verlegungen) der verlegten Neugeborenen zu Kliniken außerhalb des LMU Klinikums transportiert. Die Abb. 5 zeigt das Verlegungsaufkommen aller Standorte als Monatssumme im Zeitverlauf. Der lineare Trend illustriert hierbei ein steigendes Verlegungsaufkommen im Beobachtungszeitraum, wenn auch der Kurvenverlauf im Zeitraum stark schwankt.

### 2-stufige Regressionsanalyse des Verlegungsaufkommens

Durch die lineare Regressionsanalyse konnte gemäß *Zusatztabelle 2* ein auf dem 5 %-Niveau signifikanter Einfluss der pPQ auf die Bettenauslastung modelliert werden. Es zeigt sich eine negative Wirkbeziehung, sodass eine Reduktion der pPQ unter sonst gleichen Bedingungen mit einer Erhöhung der Bettenauslastung einherging. Auch die Anzahl versorgter Neugeborener beeinflusste die Bettenauslastung, wobei dieser Effekt etwas stärker war. Das Modell zeigt zusätzlich eine signifikant höhere Bettenauslastung der *Stationen NIPS* und *MIPS* im Vergleich zur *Referenzstation I10B*. Die Regressionskonstante gibt einen theoretischen Wert für die Bettenauslastung der *Referenzstation I10B* an, wenn die pPQ und die Anzahl versorgter Neugeborener den Wert Null annehmen würden.

Der Einfluss der Bettenauslastung auf das Verlegungsaufkommen wiederum wurde durch eine Negativ-Binomialregression berechnet und ist in *Zusatztabelle 3* dargestellt. Die Kovariablen zur Berücksichtigung der zeitlichen Abhängigkeit waren auf dem 5 %-Niveau nicht signifikant, sodass diesem Modell unterstellt werden darf, dass kein Einfluss durch eine Autokorrelation der Zeitreihendaten besteht. Es konnte somit eine signifikante Assoziation zwischen der Bettenauslastung und dem Aufkommen an Neugeborenen-Intensivverlegungen festgestellt werden. Eine angenommene Reduktion der Bettenauslastung um 10 % auf der *Referenzstation I10B* resultierte in einem durchschnittlichen relativen Risiko über $\exp \left(-0{,}1*2{,}5741\right)=0{,}7731$ für eine Neugeborenen-Intensivverlegung. Für eine Intensivverlegung von der *Referenzstation I10B* erhält man daher ein im Mittel um 22,69 % niedrigeres Risiko. Die Regressionskonstante gibt hier einen theoretischen Wert für das logarithmierte Verlegungsaufkommen der *Referenzstation I10B* an, wenn die Bettenauslastung bei 0 % liegen würde. Zudem ist ersichtlich, dass die *Station NIPS* grundsätzlich ein signifikant niedrigeres Verlegungsaufkommen aufweist als die *Referenzstation I10B*. Dieser Unterschied im Verlegungsaufkommen war für die *Station MIPS* nicht signifikant.

Diese beiden dargestellten Modelle dürfen unter sonst gleichen Bedingungen verknüpft werden. Wie in der deskriptiven Statistik geschildert, liegt die mittlere pPQ aller Standorte bei rund 79 %. Geht man hypothetisch davon aus, dass man die pPQ im Mittel um 21 % auf einen Wert von 100 % erhöhen kann, was einer Vollbesetzung der Pflegepersonalstellen entspräche, würde die Bettenauslastung der *Referenzstation I10B* gemäß der *Zusatztabelle 2* im Mittel um 9,77 % sinken. Dies würde entsprechend der *Zusatztabelle 3* dazu führen, dass das durchschnittliche relative Risiko einer Neugeborenen-Intensivverlegung aus der *Referenzstation I10B* durchschnittlich um 22,23 % (RR = 0,7777) sinkt. Das bedeutet zudem, dass durch diese Optimierung der pPQ unter Aufrechterhaltung aller Rahmenbedingungen rund alle 5 Tage (*Number Needed to Treat – *NNT = 4,5) eine Intensivverlegung verhindert werden könnte. Der Zusammenhang zwischen der rechnerischen Veränderung der pPQ und dem relativen Risiko für eine Neugeborenen-Intensivverlegung sowie die daraus errechnete NNT sind der *Zusatzabbildung 12* zu entnehmen.

Einen signifikanten direkten Zusammenhang zwischen der pPQ und dem Verlegungsaufkommen konnten wir durch die Negativ-Binomialregression hingegen nicht modellieren. Des Weiteren bestand kein signifikanter Einfluss der rPQ auf das Verlegungsaufkommen.

## Diskussion

Diese Studie untersucht exemplarisch erstmals anhand standardisiert erhobener Daten den Pflegepersonalmangel auf neonatologischen Intensivstationen und dessen Auswirkungen auf die pflegerische Versorgungsqualität im Sinne der QFR-RL, die Versorgungskapazität und die daraus resultierende Versorgungssicherheit der Bevölkerung. Hierbei konnte ein gravierender planerischer Pflegepersonalmangel auf den neonatologischen Intensivstationen des LMU Klinikums nachgewiesen werden, der zu einer deutlichen Reduktion der Versorgungskapazitäten führte. Im Verlauf des Beobachtungszeitraums 08/2017–05/2021 war ein Anstieg der Abmeldestunden der Stationen zu beobachten. Zudem konnte ein signifikanter Anstieg der Bettenauslastung betreibbarer Betten infolge der personellen Unterbesetzung dargestellt werden, wodurch das relative Risiko der versorgten Neugeborenen für eine postnatale Interhospitalverlegung anstieg. Durch eine Erhöhung der besetzten Pflegepersonalstellen könnte wiederum die Auslastung reduziert und im Weiteren Neugeborenen-Intensivverlegungen verhindert werden.

### Kapazitätsengpässe und Mehrbelastung des Pflegepersonals führen zur Verschärfung

Im Rahmen der deskriptiven Analyse zeigte sich, dass der Mittelwert der realen Personalerfüllungsquote (rPQ) deutlich höher lag als die mittlere planerische Personalerfüllungsquote (pPQ). Diese Diskrepanz könnte so interpretiert werden, dass die Versorgung der Neugeborenen trotz des Pflegepersonalmangels gesichert ist. Dies ist im Wesentlichen für die bereits aufgenommenen Patient*innen zutreffend, wohingegen die Versorgungssicherheit im Sinne der Gewährleistung einer ausreichenden Versorgungskapazität für die Bevölkerung jedoch deutlich gefährdet ist. In unseren Daten ist die qualitätssichernde Wirkung der QFR-RL durch die hohen rPQ-Werte durchaus zu erkennen, was vermutlich die fehlende statistisch signifikante Assoziation zwischen der rPQ und dem Verlegungsaufkommen erklärt. Allerdings kann diese Versorgungsqualität nur durch Anpassungen der Versorgungskapazität und einer höheren Beanspruchung des eingesetzten Pflegepersonals erreicht werden.

Ein ähnliches Bild zeichnet die zeitliche Entwicklung der Ergebnisqualitätsindikatoren, wie z. B. der Qualitätsindex zur Versorgung Frühgeborener der Bayerischen Perinatal- und Neonatalerhebung [25, 26]. Dieser Qualitätsparameter für die Versorgung hat sich über den Beobachtungszeitraum für die Perinatalzentren des LMU Klinikums ebenfalls nicht verschlechtert. Somit kann davon ausgegangen werden, dass hier die Versorgungsqualität bereits stationärer Neugeborener gesichert ist. Der Versorgungskapazitätsmangel verhindert jedoch Neuaufnahmen und führt zu vermehrten Abmeldestunden von der Bevölkerungsversorgung. Die Neugeborenen müssen allerdings im Umkehrschluss bei unvermeidbaren Aufnahmen, wie Zwangsbelegungen oder Notfällen, und nicht ausreichender Bettenkapazität einem eigentlich zu vermeidenden Transportrisiko ausgesetzt werden.

Unter Berücksichtigung des demografischen Wandels [27] sowie der zusätzlichen Belastung der Pflegekräfte unter anderem durch z. B. saisonale RSV- oder SARS-CoV-2-Wellen [4] ist daher zu vermuten, dass es zu einer weiteren Verschärfung des Pflegepersonalmangels [18] und damit zu einer zunehmenden Unterversorgung der Bevölkerung mit neonatologischer Bettenkapazität kommt [28]. Zu bedenken ist zudem, dass durch die physisch vorhandenen Betten einer neonatologischen Intensivstation auch medizinisches Gerät vorgehalten werden muss. Ein Nichtbetreiben dieser Ausstattung führt durch die fehlende Refinanzierung zu einer zusätzlichen ökonomischen Belastung in Zeiten, in denen die Kliniken ohnehin unter enormem Kostendruck stehen [18]. Die rPQ allein zeichnet somit ein unvollständiges Bild über die pflegerische Versorgung der Einzelpatient*innen und lässt die Versorgungssicherheit im Sinne der Gewährleistung einer ausreichenden Behandlungskapazität für die Bevölkerung außer Acht [29].

Lösungsansätze wie politische Beschlüsse und die zeitnahe Umsetzung von Aktionsplänen zur Schaffung besserer Rahmenbedingungen des Pflegeberufs sowie ein kontinuierliches Monitoring der Pflege- und Versorgungssituation der Kliniken sollten als abgeleitete Maßnahmen diskutiert werden [4, 18]. Zudem wäre zu diskutieren, ob das Qualitätsverständnis im Rahmen der QFR-RL überdacht und durch Angaben zur Leistungs- und Aufnahmefähigkeit der Stationen ergänzt werden sollte.

In diesem Zusammenhang bleibt zudem abzuwarten, ob durch die Anwendung der sich im Gesetzesentwurf befindlichen Pflegepersonalregelung bei der stationären Behandlung von Kindern (Kinder-PPR 2.0) sich eine exakte und wissenschaftlich evaluierte Bestimmung des Pflegepersonalaufwandes etabliert [30]. Des Weiteren ist zu befürchten, dass die vom G‑BA für das Jahr 2024 geplante Erhöhung der Mindestmengen auf 25 versorgte Frühgeborene mit einem Geburtsgewicht von weniger als 1250 g pro Jahr [31] zu Schließungen von Perinatalzentren und damit zu einer weiteren Reduktion betreibbarer Betten führt. Dies wäre nicht zuletzt im Sinne der Versorgungssicherheit von Neugeborenen kritisch und könnte zu einer weiteren Verschärfung des Pflegepersonalmangels führen, da nicht anzunehmen ist, dass die hierdurch freigewordenen Pflegekräfte automatisch der Zentralisierung von Perinatalzentren folgen.

### Steigendes Aufkommen an Intensivverlegungen von Neugeborenen

Die vorgelegte Untersuchung hat gezeigt, dass Intensivverlegungen von Neugeborenen an den untersuchten Standorten zunehmend zur Versorgungsrealität geworden sind. Diese Folge der Kapazitätsengpässe aufgrund des Pflegepersonalmangels sollte in Anbetracht des Risikos für die Neugeborenen durch eine Verlegung neu bewertet werden. In der Regel werden im Falle eines Kapazitätsengpasses die stabilsten Kinder verlegt, wodurch sich das Transportrisiko im Vergleich zu einer unmittelbar postpartalen Verlegung deutlich reduzieren sollte. Studien zum langfristigen Outcome elektiver Verlegungen liegen jedoch nicht in ausreichender Form vor und hängen von zahlreichen Einflussfaktoren ab. Unstrittig ist allerdings, dass Neugeborene vermeidbaren physikalischen Stressoren, wie z. B. Lärm, ausgesetzt werden [32]. Ferner können sie nach Transport eine hämodynamische und immunologische Stressreaktion entwickeln [33].

In dieser Untersuchung hat sich auch gezeigt, dass der überwiegende Teil der Verlegungen innerhalb der 3 Standorte des LMU Klinikums durchgeführt wurde, weshalb vermutet werden kann, dass ein erheblicher Anteil der Verlegungen verhindert werden könnte, wenn die 3 dezentralen Standorte zu einem großen Standort zusammengefasst wären. Dieser Aspekt wird aktuell in der Umsetzung eines zukünftigen Perinatalzentrums des LMU Klinikums berücksichtigt. Ein wichtiger Faktor ist darüber hinaus, dass in Deutschland rettungsdienstliche Strukturen für den Intensivtransport von Neugeborenen fehlen, die im Erwachsenenbereich schon lange selbstverständlich sind [13]. Dabei darf zudem nicht gänzlich in den Hintergrund treten, dass ein erhöhtes neonatologisches Verlegungsaufkommen mit einer weiteren Belastung der knappen öffentlichen Rettungsdienststrukturen einhergeht, sodass eine Auswirkung auf die Notfallversorgung der Bevölkerung zu vermuten ist [34, 35].

### Limitationen

Diese Studie liefert Erkenntnisse zur strukturellen Versorgungs- und Pflegepersonalsituation eines bayerischen Maximalversorgers. Festzuhalten ist, dass eine Übertragbarkeit der Studienergebnisse auf andere Perinatalzentren durch die alleinige Betrachtung der Perinatalzentren Level I des LMU Klinikums mit ihren strukturellen Besonderheiten im Sinne der 3 relativ kleinen Einheiten nur eingeschränkt möglich ist. Hervorzuheben ist außerdem, dass in dieser Studie die Verlegungsgründe nicht differenziert betrachtet wurden, sodass auch Verlegungen enthalten sein können, welche potenziell medizinisch begründet sind. Die niedrigen Effektstärken zur Beschreibung des Verlegungsrisikos deuten daher darauf hin, dass weitere Informationen fehlen.

Ergänzend ist festzustellen, dass die vorliegenden Datenquellen für unsere Analysen nicht ideal sind und sich nur schwer verknüpfen lassen. Prospektive Analysen sollten daher durch ganzheitliche Erhebungen verbessert werden. Während es sich bei den Daten der QFR-RL um Dokumentationsdaten der einzelnen Einrichtungen handelt, welche fehlerbehaftet sein können, war bei den Personalplanwerten problematisch, dass sie nur monatsbezogen vorhanden waren. Genauere Daten zur Versorgungs- und Pflegepersonallage in Verbindung mit der Kapazitätssituation einzelner Abteilungen wären daher besser geeignet, um die tatsächliche Versorgungssituation unter den gegebenen Umständen zu bewerten.

## Fazit

Dem Mangel an Pflegepersonal auf neonatologischen Intensivstationen muss derzeit entweder durch Zugeständnisse an die Versorgungsqualität oder durch die Reduzierung der Versorgungskapazität Rechnung getragen werden. Die strengen gesetzlichen Personalvorgaben der QFR-RL sorgen allerdings dafür, dass überproportional viele Betten gesperrt oder das vorhandene Pflegepersonal im Fall von nicht planbaren Notfällen überbelastet werden muss. Diese Mangelsituation führt zu gravierenden Konsequenzen, wie z. B. vermehrten Abmeldungen von Versorgungsstrukturen und dem Risiko für eine Intensivverlegung von vulnerablen Gruppen. Die Versorgungssicherheit im Sinne der Vorhaltung ausreichender Behandlungskapazitäten für die Bevölkerung wird damit zunehmend aufs Spiel gesetzt. Daher ist festzustellen, dass sich die zu Beginn der Untersuchung vermutete Studienhypothese durch die dargestellten Ergebnisse bestätigt hat. Diesem sich in der Zukunft vergrößernden Versorgungsdefizit muss dringend mithilfe gesundheitspolitischer Strategien und eines umfassenden Monitorings begegnet werden.

## Supplementary Information




