# Supplementary material for: Does the nursing shortage in neonatal intensive care units (NICU) threaten the warranty of healthcare for newborns? The “Mary and Joseph” project
Source: Bundesgesundheitsblatt Gesundheitsforschung Gesundheitsschutz. 2023 Aug 10;66(9):1019–29. [Article in German] doi: 10.1007/s00103-023-03749-6 (PMC10465400; doi:10.1007/s00103-023-03749-6)
Supplement: Supplementary file 1 [file 103_2023_3749_MOESM1_ESM.pdf]

Onlinematerial zur Originalarbeit

Gefährdet der Pflegepersonalmangel auf neonatologischen Intensivstationen die Versorgungssicherheit Neugeborener?

Das Projekt „Maria und Josef"

Weitere deskriptive Statistiken

Im Folgenden werden weitere deskriptive Statistiken sowie weitere Abbildungen zur Verdeutlichung der im Artikel illustrierten Ergebnisse aufgezeigt.

Verlegungsaufkommen

In *Zusatzabbildung 1* ist ein Histogramm zum Verlegungsaufkommen dargestellt. Hierbei wurde eine ganzheitliche Betrachtung der neonatologischen Intensivstationen des LMU Klinikums als Perinatalzentrum Level I vorgenommen. Daher sind 1400 Tage mit Tagessummen der 3 Stationen eingegangen.

Zusatzabbildung 1: Histogramm des Verlegungsaufkommens pro Tag an allen Standorten[a] (*eigene Abbildung*)

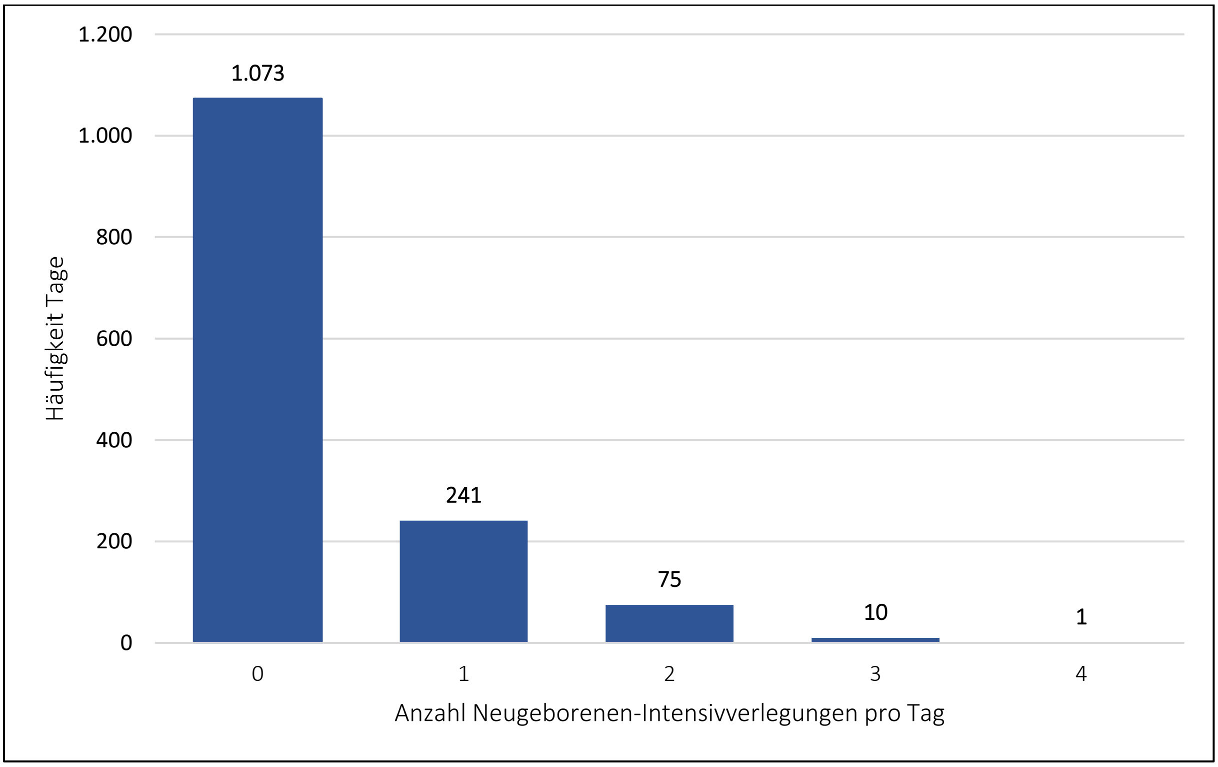

[a]**Datengrundlage**: Rettungsdienstliche Einsatzdaten

Es ist zu erkennen, dass die meisten Tage im Beobachtungszeitraum kein Aufkommen an Neugeborenen-Intensivverlegungen aufweisen. Im Maximum wurden bis zu 4 Verlegungen an einem Tag durchgeführt. Zudem ist anhand des Histogramms der Verteilungscharakter der Zielvariable für die Regressionsmodelle aufgezeigt. Näheres hierzu ist im Abschnitt *Weitere Hintergründe der Regressionsanalyse* zu finden.

Zusatzabbildung 2: Verlegungsaufkommen pro Tag als Monatsmittel im Zeitverlauf je Station[a] (*eigene Abbildung*)

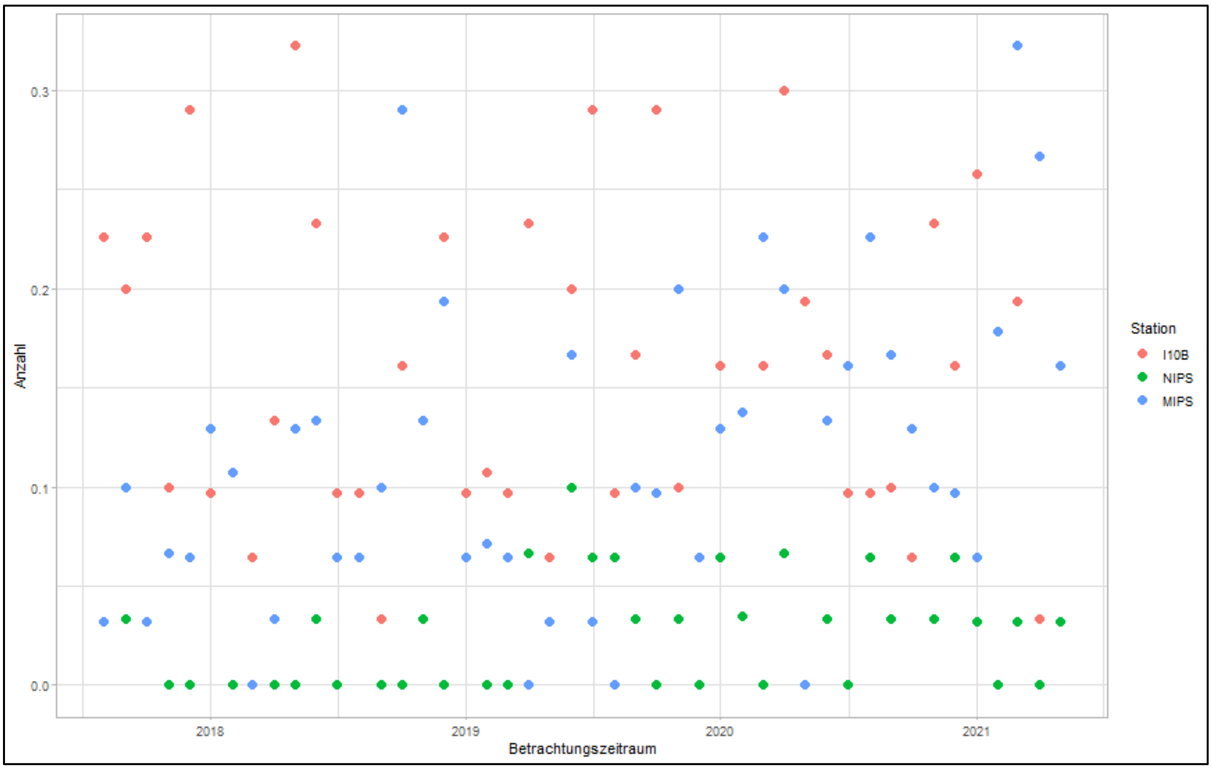

[a]**Datengrundlage**: Rettungsdienstliche Einsatzdaten

Die *Zusatzabbildung 2* stellt im Weiteren das Verlegungsaufkommen im Monatsmittel je Station im Zeitverlauf dar. Dabei ist zu sehen, dass die wenigsten Monate gar keine Verlegungen aufweisen. Auf der *Station I10B* kommt dies im Betrachtungszeitraum nicht vor.

Anzahl versorgter Neugeborener

Wie im Artikel beschrieben, ist die Anzahl versorgter Neugeborener im Zeitverlauf gesunken. Dies kann der *Zusatzabbildung 3* entnommen werden. Der beschriebene negative Trend ist am deutlichsten auf der *Station I10B* zu erkennen. Zudem ist festzustellen, dass die *Station I10B* in den meisten Monaten im Mittel die meisten Neugeborenen versorgt hat. Mit Hinblick auf *Zusatzabbildung 7* ist dies damit zu erklären, dass diese Station die meisten aufstellbaren Betten aufweist.

Zusatzabbildung 3: Anzahl versorgter Neugeborener pro Tag als Monatsmittel im Zeitverlauf[a] (*eigene Abbildung*)

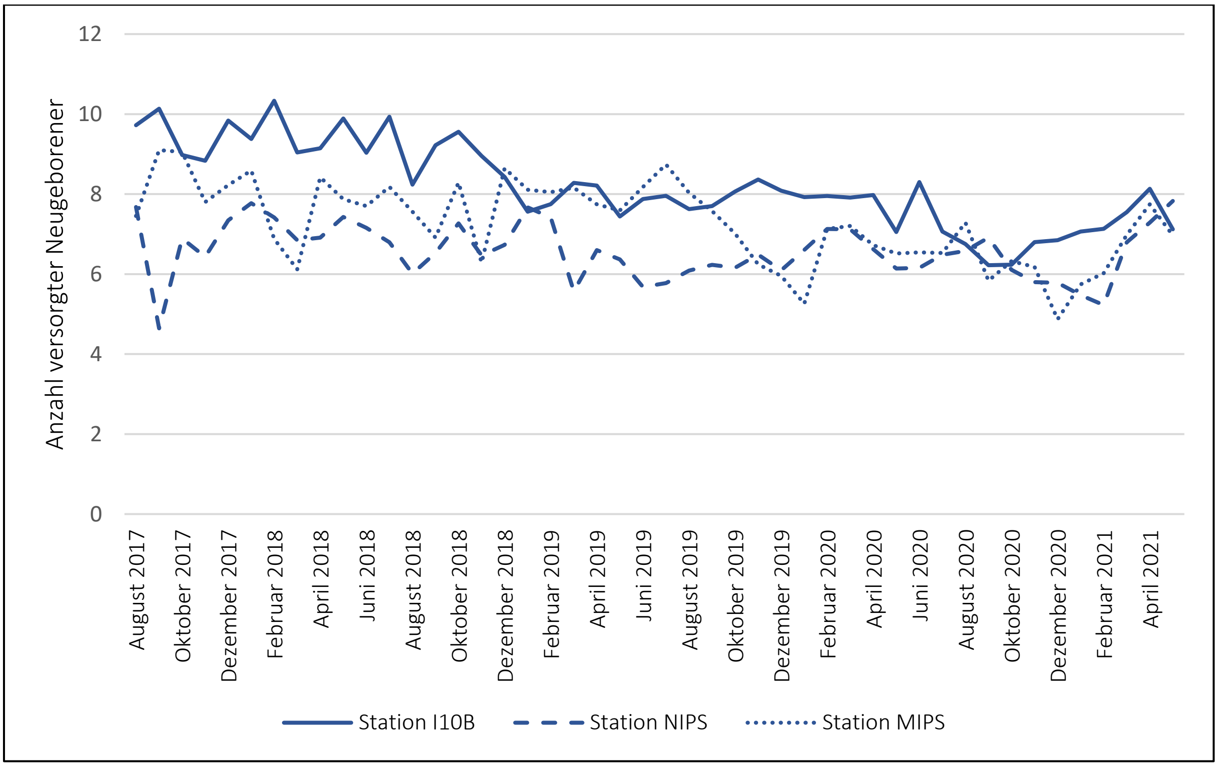

[a]**Datengrundlage**: Daten gemäß Qualitätssicherungs-Richtlinie Früh- und Reifgeborene (QFR-RL)

Diese negative Entwicklung kann unter anderem darauf zurückgeführt werden, dass im Zeitverlauf in Anbetracht einer hohen Bettenauslastung auch weniger Betten betreibbar waren. Näheres hierzu wird im Punkt *Bettenstruktur* ausgeführt. Dadurch ist die Reduktion der Versorgungskapazität auch in der absoluten Anzahl versorgter Neugeborener zu erkennen.

Planerische Personalerfüllungsquote (pPQ)

Die pPQ gibt den Anteil der besetzten Vollkosten-Pflegepersonalstellen (VK-Stellen) an den benötigten VK-Stellen zur personellen Bewerkstelligung der aufstellbaren Betten an. Die Anzahl der benötigten VK-Stellen ist durch die prospektive Pflegepersonalkalkulation errechnet und geht daher von der Annahme aus, dass alle aufstellbaren Betten versorgt werden können. Die beschriebenen pPQ-Werte werden in *Zusatzabbildung 4* verdeutlicht. Hierbei wird die absolute Anzahl benötigter und vorhandener VK-Stellen je Station als Monatsmittel im Zeitverlauf dargestellt. Die Anzahl benötigter VK-Stellen sind anhand der grauen Linien grafisch verdeutlicht. Da diese im Rahmen des Pflegepersonalmanagements auf Grundlage der Anzahl physisch aufstellbarer Betten berechnet werden, bleiben die benötigten VK-Stellen weitgehend konstant.

Zusatzabbildung 4: Planerisch notwendige und besetzte Anzahl Vollkosten-Pflegepersonalstellen (VK-Stellen) als Monatsmittel im Zeitverlauf[a] (*eigene Abbildung*)

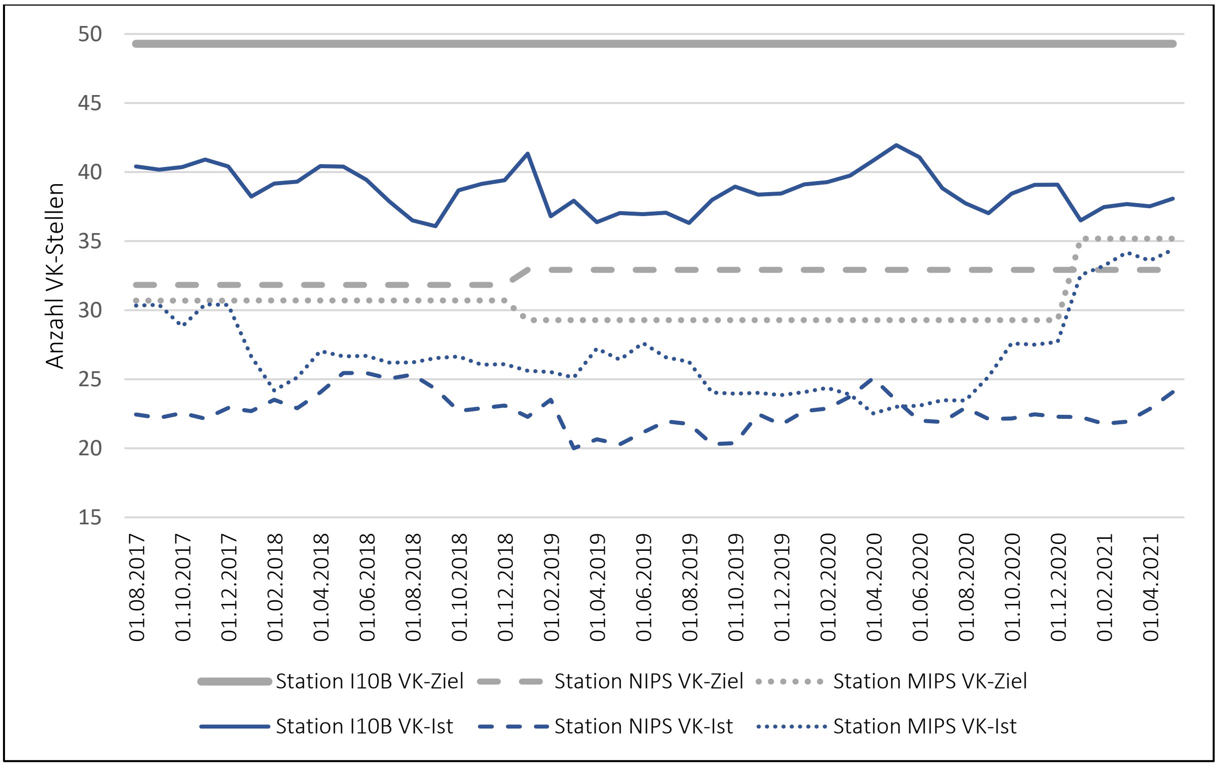

[a]**Datengrundlage**: Personalplanwerte

Anmerkungen: VK-Ziel = Anzahl planerisch notwendiger Vollkosten-Pflegepersonalstellen zur Bewerkstelligung der aufstellbaren Betten, VK-Ist = Anzahl besetzter Vollkosten-Pflegepersonalstellen

Es wird anhand dieser Darstellung nochmals die grundsätzliche Diskrepanz und das Verhältnis zwischen benötigten und besetzten VK-Stellen der neonatologischen Intensivstationen deutlich und die Kontinuität des Problems aufgezeigt.

Reale Personalerfüllungsquote (rPQ)

Die rPQ gibt den Anteil der eingesetzten Pflegekräfte an den notwendigen Pflegekräften zur Versorgung der tatsächlich vorhandenen Neugeborenen an. Eine rPQ von 100 % sagt aus, dass im Tagesmittel genauso viele Pflegekräfte eingesetzt wurden, wie zur Versorgung der Neugeborenen gemäß der Qualitätsanforderungen der „Qualitätssicherungs-Richtlinie Früh- und Reifgeborene“ (QFR-RL) des Gemeinsamen Bundesausschusses notwendig war. Allerdings ist festzuhalten, dass dieser Parameter sehr anfällig für Fluktuationen der Anzahl versorgter Neugeborener ist. Kommt es zu einer plötzlichen Erhöhung der Neugeborenen (z. B. aufgrund von Zuverlegungen oder durch eine Mehrlingsgeburt), kann aufgrund dienstplanerischer und arbeitsrechtlicher Erfordernisse meist nicht unmittelbar mehr Pflegepersonal eingesetzt werden, woraus dann niedrigere rPQ-Werte resultieren und umgekehrt.

Die *Abbildung 2* des Artikels zeigt, dass die rPQ im Monatsmittel auf allen Stationen im Mittel zwischen 80 % und 100 % schwankt. Bemisst sich die rPQ auf über 100 %, sagt dies eine personelle Überbesetzung gemessen an den Pflegeschlüsseln der versorgten Neugeborenen gemäß QFR-RL aus. Allerdings weisen die meisten Monate eine im Mittel messbare personelle Unterbesetzung auf. In den letzten Monaten des Beobachtungszeitraums ist eine steigende reale Pflegepersonalbesetzung zu erkennen.

In die *Zusatzabbildung 5* gehen die 1400 rPQ-Werte als Tagesmittelwert aus 3 Schichten pro Tag je Station ein. Hierbei ist hervorzuheben, dass die *Station NIPS* an rund 75 % der Beobachtungstage eine personelle Unterbesetzung zu verzeichnen hat. Ein ähnliches Bild zeichnet sich bei den anderen Stationen ab, wobei die Größenordnungen geringer sind.

Zusatzabbildung 5: Boxplot der realen Personalerfüllungsquote pro Tag[a] je Station[b] (*eigene Abbildung*)

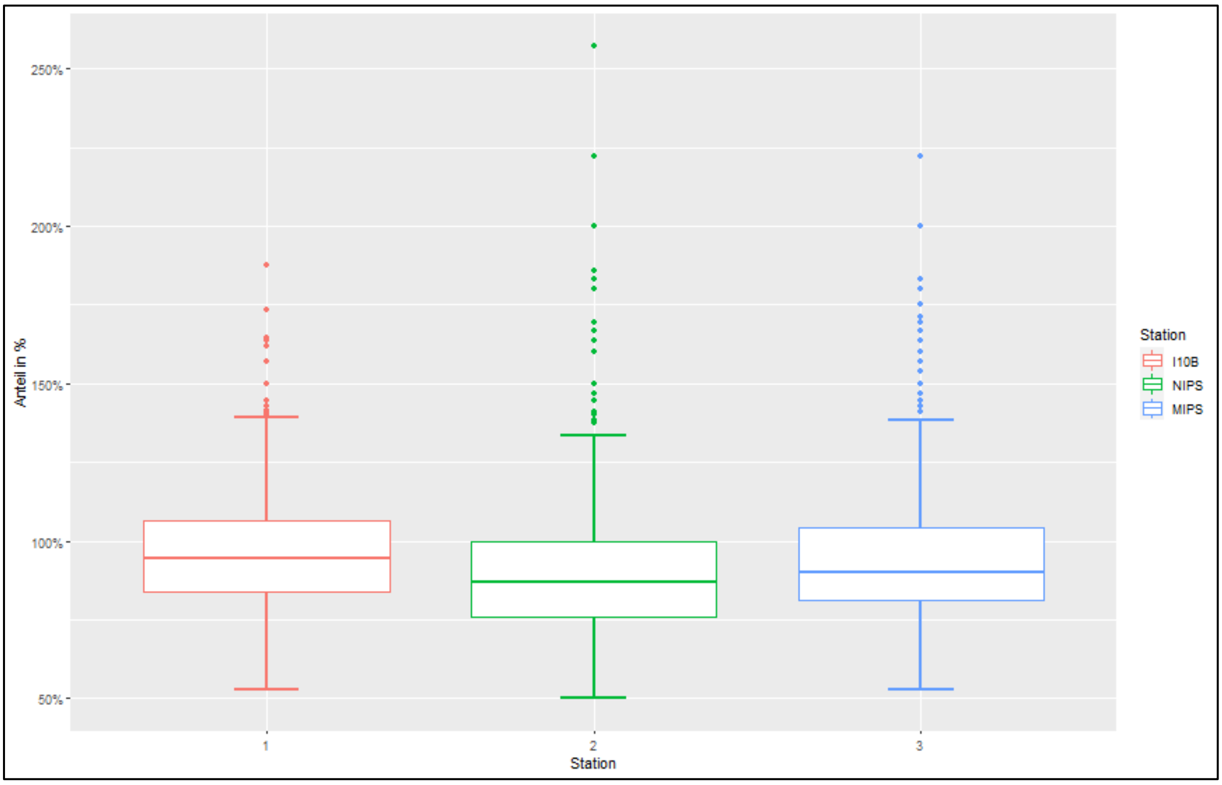

[a]Tagesmittelwert über 3 Schichten

[b]**Datengrundlage**: Daten gemäß Qualitätssicherungs-Richtlinie Früh- und Reifgeborene (QFR-RL)

Bettenstruktur

Anhand der Bettenauswertungen der neonatologischen Intensivstationen des LMU Klinikums kann die Bettenstruktur im Zeitverlauf gut untersucht werden. Der Parameter *Bettenauslastung* beschreibt den Anteil betreibbarer Betten, der mit Neugeborenen belegt ist. Daher verknüpft dieser Parameter die QFR-RL-Daten mit den Daten aus der Bettenauswertung. Die *Zusatzabbildung 6* zeigt den Verlauf der Bettenauslastung je Station als Monatsmittel auf. Auffallend ist die Auslastungssteigerung der *Station I10B* in den letzten Monaten des Beobachtungszeitraums. Auf der *Station I10B* und *NIPS* kommt es zudem im Monatsmittel zwischenzeitlich zu einer Überbelegung der Station.

Zusatzabbildung 6: Bettenauslastung als Monatsmittel im Zeitverlauf[a] (*eigene Abbildung*)

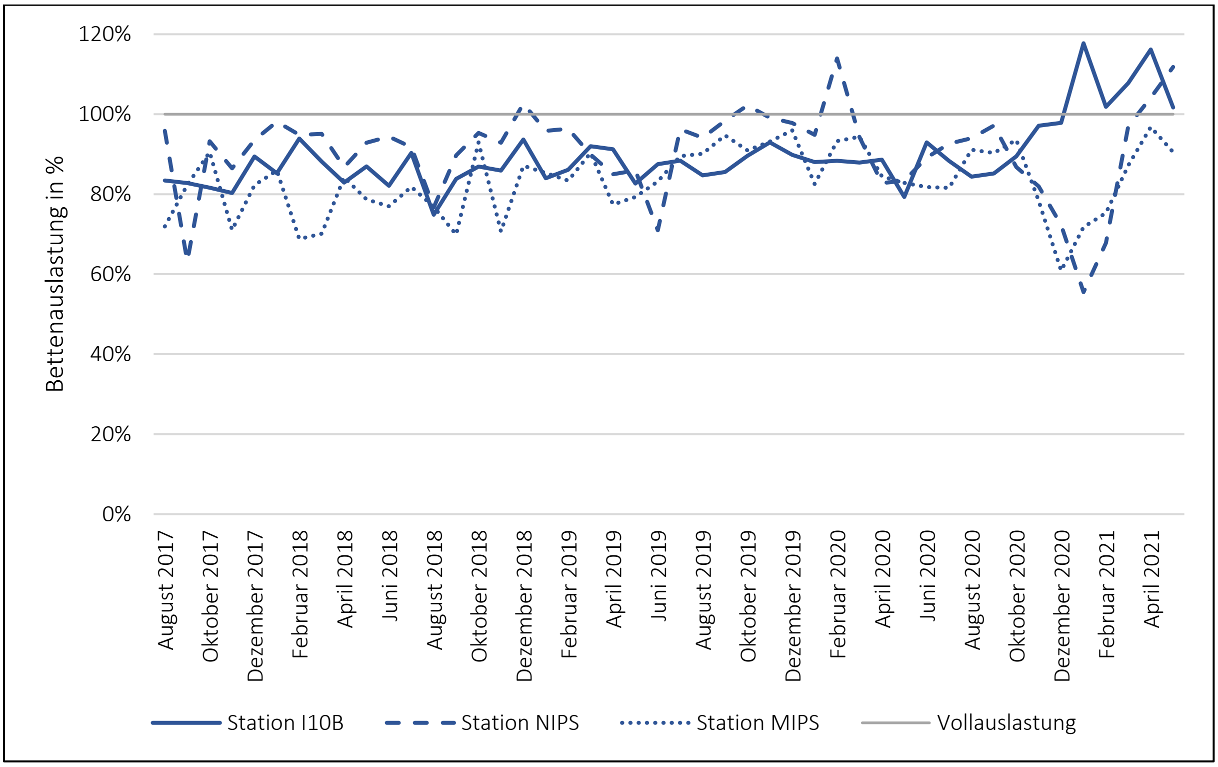

[a]**Datengrundlage**: Daten gemäß Qualitätssicherungs-Richtlinie Früh- und Reifgeborene (QFR-RL) und Bettenauswertung

Im Weiteren ist in *Zusatzabbildung 7* die absolute Anzahl an aufstellbaren und betreibbaren Betten als Monatsmittel je Station im Zeitverlauf abgebildet. Die grauen Linien zeigen dabei die physisch aufstellbaren Betten und die blauen Linien die tatsächlich betreibbaren Betten je Station auf. Es wird deutlich, dass die aufstellbaren Betten über den Beobachtungszeitraum weitestgehend konstant bleiben. Eine Änderung der Anzahl an aufstellbaren Betten hängt dabei meist mit strukturellen Änderungen der Station zusammen. Es wird zudem deutlich, dass auf allen Stationen deutlich weniger Betten betreibbar waren. Die Anzahl an betreibbaren Betten sinkt im Verlauf insbesondere auf der *Station I10B*. Die *Abbildung 3* des Artikels stellt in Anlehnung dieser Berechnungen den Anteil der betreibbaren Betten an den aufstellbaren Betten dar, um auch einen Stationsvergleich zu ermöglichen.

Zusatzabbildung 7: Anzahl aufstellbare und betreibbare Betten als Monatsmittel im Zeitverlauf[a] (*eigene Abbildung*)

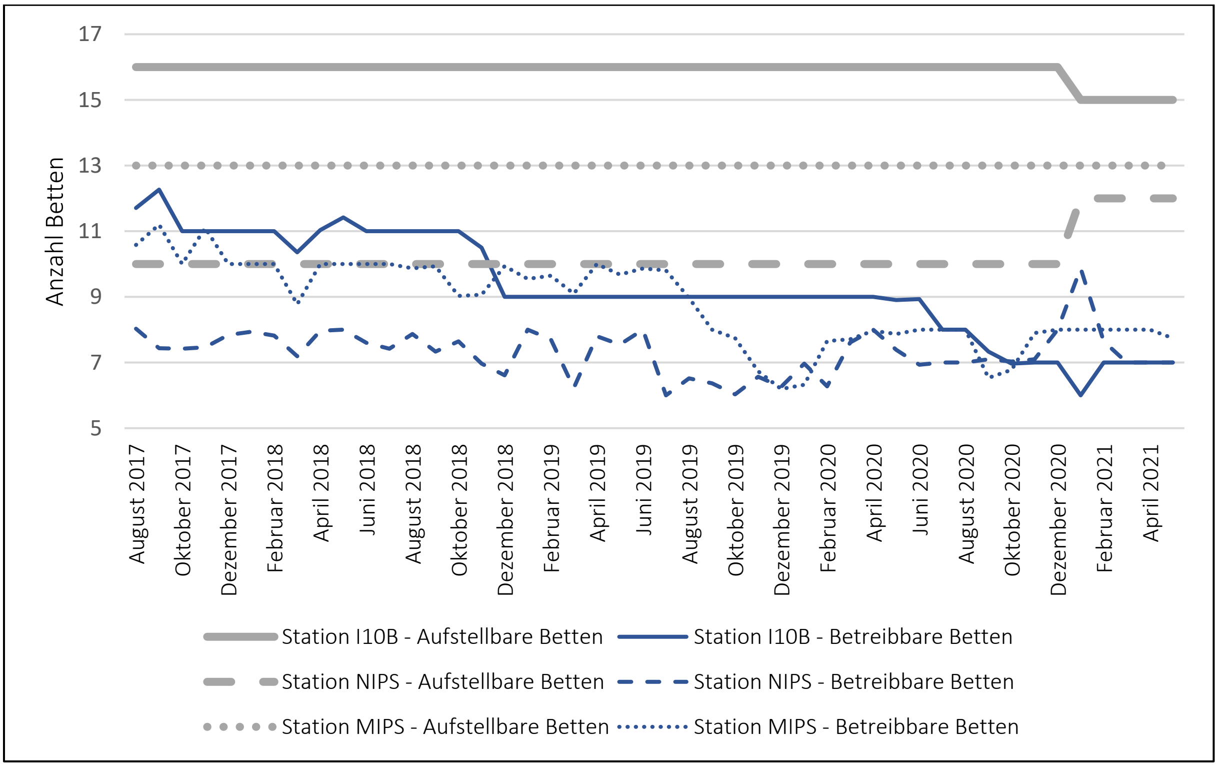

[a]**Datengrundlage**: Bettenauswertung

In *Zusatzabbildung 8* ist die absolute Anzahl aufgrund des Pflegepersonalmangels gesperrter Betten im zeitlichen Verlauf als Monatsmittel dargestellt. Betrachtet man die insgesamt gesperrten Betten und die gesperrten Betten aufgrund des Pflegepersonalmangels, stellt man zudem fest, dass fast alle gesperrten Betten im Beobachtungszeitraum auf den Pflegepersonalmangel zurückzuführen sind.

Zusatzabbildung 8: Anzahl gesperrter Betten aufgrund Pflegepersonalmangel als Monatsmittel im Zeitverlauf[a] (*eigene Abbildung*)

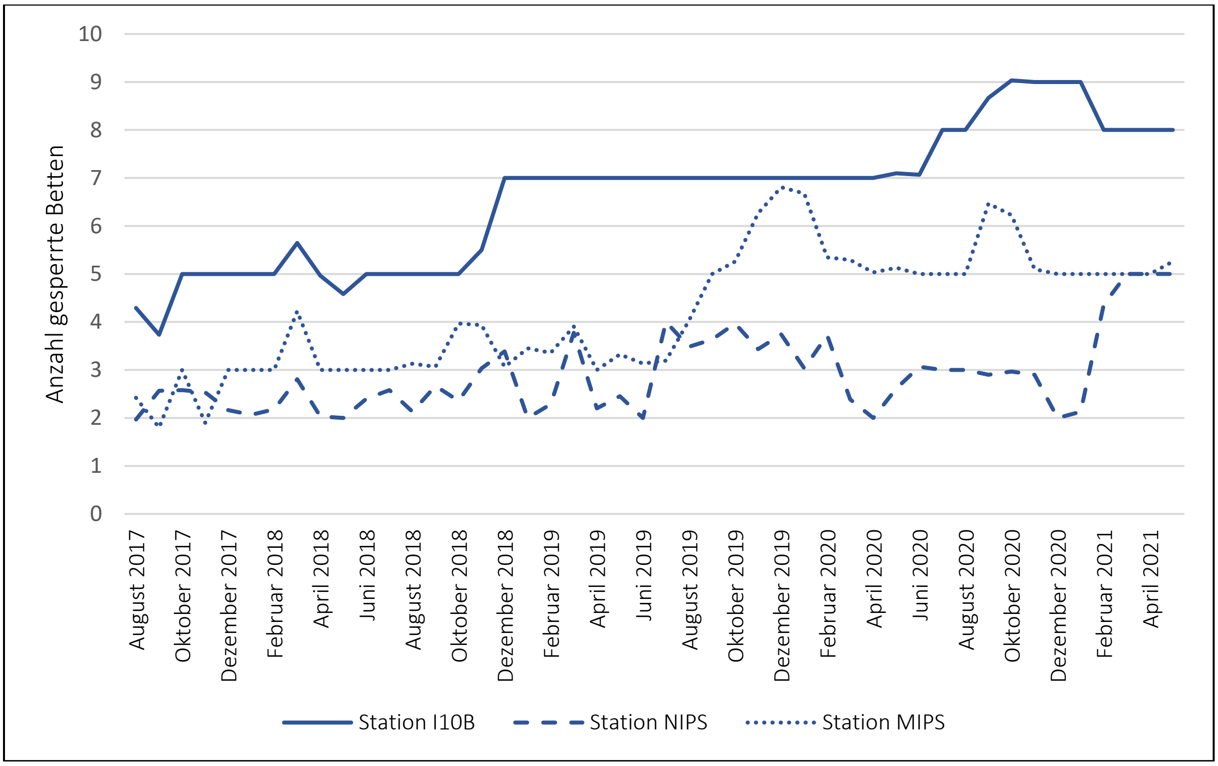

[a]**Datengrundlage**: Bettenauswertung

Abmeldung von der Regelversorgung der Bevölkerung

Um die Konsequenzen der reduzierten Versorgungskapazität auf die Versorgung der Bevölkerung neben dem Verlegungsverhalten darzustellen, ist die Entwicklung der Klinikabmeldungen relevant. Dazu wurde das Leitstellenmeldeportal „IVENA eHealth®" herangezogen. Das Abmeldeverhalten wurde hierbei durch den Anteil der abgemeldeten Versorgungstunden pro Tag gemessen und zum Ausdruck gebracht.

Über den Beobachtungszeitraum hat sich eine Zunahme der Abmeldungen von der Versorgung der Bevölkerung gezeigt. Dieses Phänomen ist als Folge des im Artikel beschriebenen Personalmangels, der zu vermehrten Bettensperrungen und erhöhter Bettenauslastung führt, zu betrachten. Da sich das Abmeldeverhalten der 3 Stationen stark unterschieden hat, eignete sich dieser Parameter nicht als Zielvariable eines Regressionsmodells. Dennoch durchgeführte Modellierungen zur Überprüfung dieser Hypothese haben zu keiner signifikanten Assoziation zwischen dem Abmeldeverhalten und dem Aufkommen an Neugeborenen-Intensivverlegungen geführt.

In *Zusatzabbildung 9* ist der Verlauf des Anteils abgemeldeter Versorgungstunden in IVENA eHealth® als Monatsmittel je Station abgetragen.

Zusatzabbildung 9: Anteil abgemeldeter Versorgungsstunden als Monatsmittel im Zeitverlauf[a] (*eigene Abbildung*)

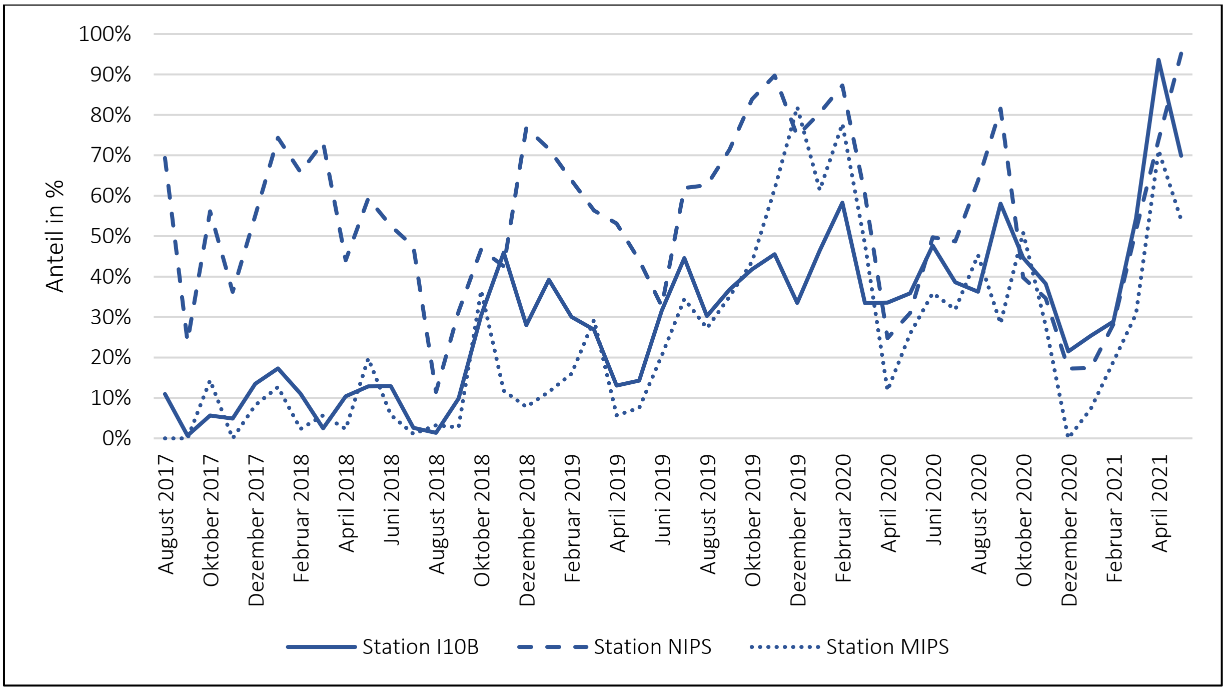

[a]**Datengrundlage**: Abmeldedaten des Leitstellenmeldeportals *IVENA eHealth®*

Vor allem die *Stationen I10B* und *MIPS* melden sich im zeitlichen Verlauf des Beobachtungszeitraums zunehmend von der Versorgung ab. Die *Station NIPS* weist bereits zu Beginn des Beobachtungszeitraums einen höheren Anteil an abgemeldeten Versorgungsstunden auf als die anderen beiden neonatologischen Intensivstationen.

In *Abbildung 3* des Artikels wurde eine ganzheitliche Betrachtung der neonatologischen Intensivstationen des LMU Klinikums vorgenommen. Dabei ist zu erkennen, dass die betrachteten Stationen neben einer zunehmenden Einschränkung ihrer Bettenkapazitäten auch ein zunehmendes Abmeldeverhalten von der Regelversorgung der Bevölkerung aufweisen. Auch wenn der Einfluss des Pflegepersonalmangels und der Bettenauslastung auf diese Variable in dieser Studie nicht näher untersucht werden konnte, würde sich dieses Ergebnis mit ähnlichen Studien zu diesem Thema decken [8]. Zu hinterfragen wäre auch, ob diese Situation auch im Zusammenhang mit Patientenablehnungen aus Kapazitätsgründen steht und damit einen direkten Einfluss auf die Bevölkerungsversorgung hat [9].

Weitere Hintergründe der Regressionsanalyse

Der Einfluss des Mangels an Pflegepersonal der neonatologischen Intensivstationen des LMU Klinikums auf das Verlegungsaufkommen wurde anhand einer zweistufigen Regressionsanalyse untersucht. Im ersten Schritt wurde die Assoziation zwischen der pPQ und der Bettenauslastung und im Weiteren zwischen der Bettenauslastung und dem Aufkommen an Neugeborenen-Intensivverlegungen analysiert. Im Folgenden werden weitere Hintergründe zu dieser Regressionsanalyse dargelegt.

Statistischer Hintergrund

Vor der Regressionsanalyse werden obligat die notwendigen Modellvoraussetzungen überprüft. Dabei ist grundlegend klarzustellen, dass bei der Nutzung von Zeitreihendaten eine Autokorrelation bzw. zeitliche Abhängigkeit vermutet werden muss und damit die Modellvoraussetzungen für lineare Modelle nicht mehr erfüllt sein können. Es ist daher anzunehmen, dass die einzelnen Tage als Beobachtungseinheit nicht als unabhängig voneinander anzusehen sind und die Standardfehler des Modells damit nicht mehr zuverlässig geschätzt werden. In letzter Konsequenz wird der Schätzparameter nicht mehr effizient berechnet und die Konfidenzintervalle falsch dargestellt. Dadurch wären keine Aussagen über die statistische Signifikanz mehr möglich. Dieser Effekt kann jedoch abhängig vom Zielparameter adjustiert werden [19].

In dieser Untersuchung wird hierfür bei einem stetigen Zielparameter, wie der Bettenauslastung, die Cochrane-Orcutt-Transformation [20] mit dem R-Paket *orcutt* für das Statistikprogramm R Studio [26] durchgeführt. Hierbei wird mittels Durbin-Watson-Test geprüft, ob eine Autokorrelation erster Ordnung besteht [19]. Die Signifikanz dieses Tests zeigt dabei das Vorliegen einer Autokorrelation erster Ordnung an. Anschließend wird das lineare Regressionsmodell automatisch durch ein iteratives Verfahren transformiert und der Durbin-Watson-Test mit diesem transformierten linearen Regressionsmodell wiederholt. War die Adjustierung der Autokorrelation erfolgreich, resultiert beim wiederholten Durbin-Watson-Test kein statistisch signifikantes Ergebnis mehr. Die Regressionskoeffizienten können dann entsprechend einem regulären linearen Regressionsmodell interpretiert werden.

Bei der Modellierung von diskreten Zielvariablen, wie dem Aufkommen an Neugeborenen-Intensivtransporten, ist eine Cochrane-Orcutt-Transformation jedoch nicht angemessen, sodass in dieser Studie die zeitliche Abhängigkeit mithilfe unabhängiger Kovariablen mitmodelliert wurde. Hierfür kam das R-Packet *tscount* zur Anwendung. Mit zusätzlichen unabhängigen Variablen *past_obs* sowie *past_mean* wird sowohl der Effekt des unabhängigen Werts der letzten Zeiteinheit als auch der Effekt des bedingten Erwartungswerts der letzten Zeiteinheit in das Modell miteinbezogen [21].

Eine weitere Herausforderung bei der geplanten Modellierung stellt die Datenstruktur dar. Wie im Abschnitt *Datengrundlage* des Artikels bereits ausgeführt, wurden die Daten der QFR-RL aufgrund der schichtbezogenen Dokumentation über einen Tagesmittelwert aufbereitet. Eine Modellierung in dieser Struktur führt allerdings zu einer Verzerrung der Assoziation. Dies liegt daran, dass eine Verlegung in der Frühschicht entsprechend zu einer Reduktion der Bettenauslastung und realen Personalerfüllungsquote in der Spät- und Nachtschicht führt. Bei Betrachtung der rettungsdienstlichen Einsatzdaten war ersichtlich, dass die meisten Verlegungen in der Frühschicht durchgeführt wurden bzw. nach Einschätzung der Leitung der Neonatologie des LMU Klinikums zumindest die Verlegungsentscheidungen in der Frühschicht auf Basis der Rahmenbedingungen getroffen wurde. Daher wird für die Modellierung bei den Daten der QFR-RL ausschließlich Werte der Frühschicht eingeschlossen.

Um adäquate Modelle zu erstellen und mögliche Confounder zu erkennen, wurden Wirkbeziehungen der verschiedenen Variablen angenommen. Diese a priori vermuteten Wirkbeziehungen, wodurch auch die Studienhypothese entstanden ist, ist der *Zusatzabbildung 10* zu entnehmen. Die Färbung bringt die jeweilige Datenquelle zum Ausdruck. Die rote Färbung verdeutlicht dabei die Bettenauswertung, die türkise Färbung die QFR-RL-Daten und die violette Färbung die rettungsdienstlichen Einsatzdaten.

Zusatzabbildung 10: Vermutetes Wirkschema der betrachteten Variablen (*eigene Abbildung*)

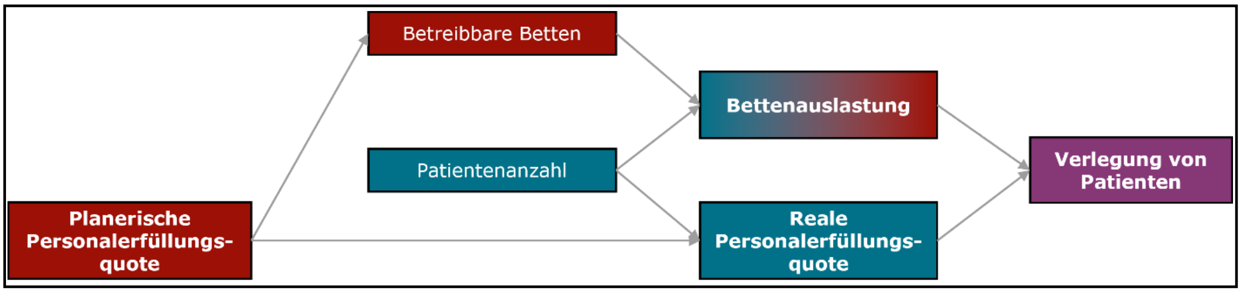

Somit war ersichtlich, dass eine Korrelation zwischen der pPQ und rPQ vermutet werden muss. Dies hat sich auch in der Betrachtung der Korrelation bestätigt. Daher konnten nicht beide Variablen in ein Regressionsmodell als unabhängige Kovariablen aufgenommen werden.

Des Weiteren war die Bestimmung der Verteilung der Zielvariablen maßgeblich. Bei Betrachtung des Histogramms des Verlegungsaufkommens in *Zusatzabbildung 1* ist grafisch eine Poisson-Verteilung bzw. eine Negativ-Binomialverteilung anzunehmen. Bei der Berechnung beider Modelle und dem

Vergleich der Log-Likelihood-, AIC- und BIC-Werte zeigte sich jedoch, dass die Negativ-Binomialregression für die vorliegenden Daten besser geeignet ist.

Diagnostik einer Autokorrelation erster Ordnung

Im Folgenden werden die Ergebnisse der Diagnose einer zeitlichen Abhängigkeit in den Zeitreihendaten durch den Durbin-Watson-Test aufgeführt. Festzuhalten ist dabei, dass der Durbin-Watson-Test lediglich im Hinblick auf die Cochrane-Orcutt-Transformation berechnet wurde und damit nur beim linearen Regressionsmodell der Bettenauslastung entsprechend der *Zusatztabelle 2* zum Tragen kam. Für das Negativ-Binomialregressionsmodell des Verlegungsaufkommens wurde eine Autokorrelation zum einen mithilfe der Signifikanz der Kovariablen *past_obs* und *past_mean* und zum anderen durch grafische Darstellungen beurteilt.

Die Nullhypothese des Durbin-Watson-Tests besagt, dass keine Autokorrelation erster Ordnung vorliegt. Entsprechend beschreibt die Alternativhypothese, dass eine Autokorrelation erster Ordnung besteht [19]. Die Ergebnisse des Tests sind in folgender *Zusatztabelle 1* zu finden.

Zusatztabelle 1: Ergebnisse des Durbin-Watson-Tests

Model	Estimation	p-Value[a]
Original	1,61773	1,841e-35
Transformed	2,07745	9,943e-01
Modell	**Abhängige Variable**: Bettenauslastung<br>**Unabhängige Variable**: pPQ	

Anmerkung: pPQ = Planerische Personalerfüllungsquote
[a]p-Wert auf dem 5 %-Signifikanzniveau

Es ist ersichtlich, dass bei der Prüfung des originalen und nicht transformierten linearen Regressionsmodells die Alternativhypothese aufgrund der Signifikanz des p-Werts angenommen werden muss. Bei Wiederholung des angepassten Regressionsmodells durch die Cochrane-Orcutt-Transformation resultiert kein signifikantes Testergebnis mehr und die Nullhypothese kann beibehalten werden. Es ist ersichtlich, dass die Adjustierung einer ursprünglich vorliegenden Autokorrelation erfolgreich war und dass das im Artikel dargestellte lineare Regressionsmodell der Bettenauslastung wieder effizient geschätzt wurde.

Die Negativ-Binomialregression gemäß der *Zusatztabelle 3* illustriert, dass die Kovariablen zur Beschreibung einer zeitlichen Abhängigkeit erster Ordnung nicht signifikant sind. Dadurch ist zu

unterstellen, dass die Zeitreihendaten in diesem Modell zu keinen autokorrelierten Störungen geführt haben. Dies bedeutet allerdings nicht, dass in diesen Zeitreihendaten keine Autokorrelation besteht, zumal der erste Durbin-Watson-Test eine Autokorrelation erster Ordnung festgestellt hat. Dennoch bestand das Ziel, den Einfluss auf die Effizienz der Modellschätzung durch die zeitliche Abhängigkeit zu verhindern, um eine zuverlässige Schätzung zu gewährleisten [19]. In diesem Zusammenhang ist auch eine grafische Beurteilung der Residuen hilfreich. In *Zusatzabbildung 11* sind die Residuen des im Artikel dargestellten Negativ-Binomialregressionsmodells des Verlegungsaufkommens im Zeitverlauf abgebildet. Dabei ist jedoch hervorzuheben, dass pro Tag je Station ein Wert in diese Abbildung eingegangen ist. Daher sind 4200 Beobachtung dargestellt, welche allerdings eine grafische Überschneidung der 3 neonatologischen Intensivstationen des LMU Klinikums über den Betrachtungszeitraum bedeuten.

Zusatzabbildung 11: Darstellung der Abweichung der vorhergesagten Werte von den tatsächlich beobachteten Werten (Residuen) im Zeitverlauf der Negativ-Binomialregression des Verlegungsaufkommens (*eigene Abbildung*)

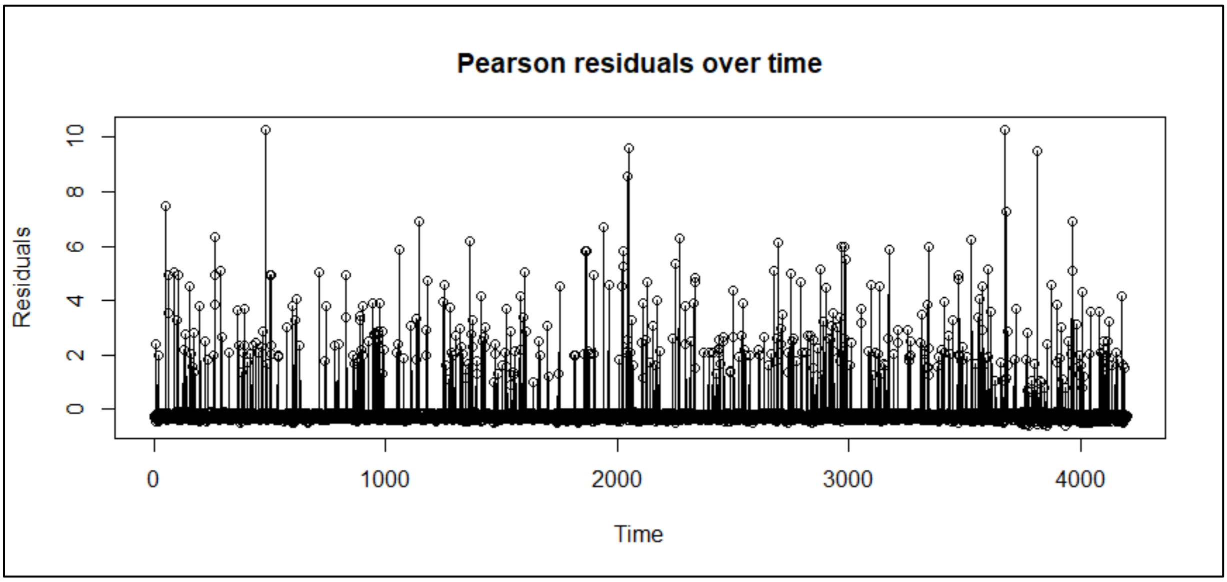

Grundsätzlich sollten die Residuen keinem systematischen Muster folgen und daher randomisiert verteilt sein. In *Zusatzabbildung 11* ist durch die Extremwerte der Residuen durchaus eine Saisonalität zu beobachten. Allerdings scheint es, dass sich diese Beobachtung lediglich auf die Extremwerte beschränkt, sodass insgesamt keine signifikante zeitliche Abhängigkeit durch die Variablen *past_obs* und *past_mean* geschätzt wurde.

Ergebnisse der Regressionsanalyse

Im Folgenden werden die detaillierten Ergebnisse der zweistufigen Regressionsanalyse in Ergänzung zu den Ausführungen des Artikels aufgeführt.

Die *Zusatztabelle 2* stellt die Regressionsergebnisse der Modellierung der Bettenauslastung durch die pPQ und der Anzahl versorgter Neugeborener dar. Diese Ergebnisse wurden anhand der Cochrane-Orcutt-Transformation von einer Autokorrelation erster Ordnung bereinigt.

Zusatztabelle 2: Lineares Regressionsmodell der Zielvariablen Bettenauslastung

Unabhängige Variable	Abhängige Variable	Standardfehler
	Bettenauslastung	
pPQ[a]	-0,465***	0,037
Anzahl versorgter Neugeborener	0,080***	0,001
Station NIPS[b]	0,105***	0,005
Station MIPS[b]	0,061***	0,006
Konstante	0,605***	0,029
Beobachtungen	4200	
R^2	0,4861	
Adjustiertes R^2	0,4858	
Standardfehler (df = 4195)	0,1194	
F (df = 3; 4195)	991,9***	

Anmerkungen:
*p < 0,1 **p < 0,05 ***p < 0,01 auf dem 5 %-Signifikanzniveau, pPQ = Planerische Personalerfüllungsquote

[a] Die pPQ wird als Anteil an 1 berechnet.

[b] Die Bettenauslastung wird anhand der *Station I10B* als Referenzstation modelliert, sodass der Auslastungsunterschied der Vergleichsstationen über die Dummy-Variablen *Station NIPS* und *Station MIPS* zum Ausdruck gebracht wird.

Die *Zusatztabelle 3* zeigt die Ergebnisse der Negativ-Binomialregression des Verlegungsaufkommens durch die Bettenauslastung auf. Dabei ist zu beachten, dass die Linkfunktion logarithmiert ist, sodass der Regressionskoeffizient der Bettenauslastung entsprechend exponiert werden muss. Daher erhält man ein relatives Risiko für eine Neugeborenen-Intensivverlegung bei entsprechender Veränderung der Bettenauslastung. Ein Beispiel wird im Zusammenhang mit *Zusatzabbildung 12* aufgezeigt.

Zusatztabelle 3: Negativ-Binomialregression der Zielvariablen Verlegungsaufkommen[a]

Unabhängige Variable	Abhängige Variable	Standardfehler	5%-Konfidenzintervall (KI)
	Verlegungsaufkommen		
Beobachtung letzter Tag (*past_obs*)	0,0507	0,249	-0,438; 0,539
Bedingter Erwartungs-wert letzter Tag (*past_mean*)	-0,1287	0,125	-0,374; 0,116
Bettenauslastung[a]	2,5741*	0,340	1,907; 3,241
Station NIPS[b]	-1,6315*	0,173	-1,970; -1,293
Station MIPS[b]	-0,3627	0,192	-0,738; 0,013
Konstante	-4,5329*	0,441	-5,398; -3,668
Beobachtungen	4200		
Log-Likelihood	-1327,338		
AIC	2668,677		
BIC	2713,077		
QIC	2691,683		
Linkfunktion	log		

Anmerkung: *Statistisch signifikant im Sinne 0 ∉ KI, AIC = Akaike-Informationskriterium, BIC = Bayes'sches Informationskriterium, QIC = Quasi-Likelihood unter Unabhängigkeitsmodellkriterium

[a] Die Bettenauslastung wird als Anteil von 1 berechnet.

[b] Das Verlegungsaufkommen wird anhand der *Station I10B* als Referenzstation modelliert, sodass der Verlegungsunterschied zu den Vergleichsstationen über die Dummy-Variablen *Station NIPS* und *Station MIPS* zum Ausdruck gebracht wird.

Zusammenhang der zweistufigen Regressionsanalyse

Im Artikel wurde dargestellt, wie die beiden Regressionsmodelle unter Beachtung der Ceteris-Paribus-Annahme verknüpft werden können. Zusammenfassend ist festzuhalten, dass ein Absinken der pPQ im Mittel zu einer Erhöhung gesperrter Betten, ausgedrückt durch die Erhöhung der Bettenauslastung, führt. Diese Einschränkung der Bettenkapazität bzw. diese Auslastungssteigerung führt bei sonst gleichen Bedingungen zu einer Erhöhung des relativen Risikos für eine Neugeborenen-Intensivverlegung. Die *Zusatzabbildung 12* zeigt, wie sich eine hypothetische Veränderung der pPQ auf das relative Risiko einer Neugeborenen-Intensivverlegung auswirkt. Auf der Sekundärachse ist zudem die Anzahl der Tage abgebildet, nachdem eine Verlegung unter Annahme des jeweiligen Szenarios verhindert werden könnte (NNT bzw. number needed to treat). Diese Betrachtung wurde anhand der *Referenzstation I10B* vorgenommen.

Zusatzabbildung 12: Verknüpfung der berechneten Regressionsmodelle[a] (*eigene Abbildung*)

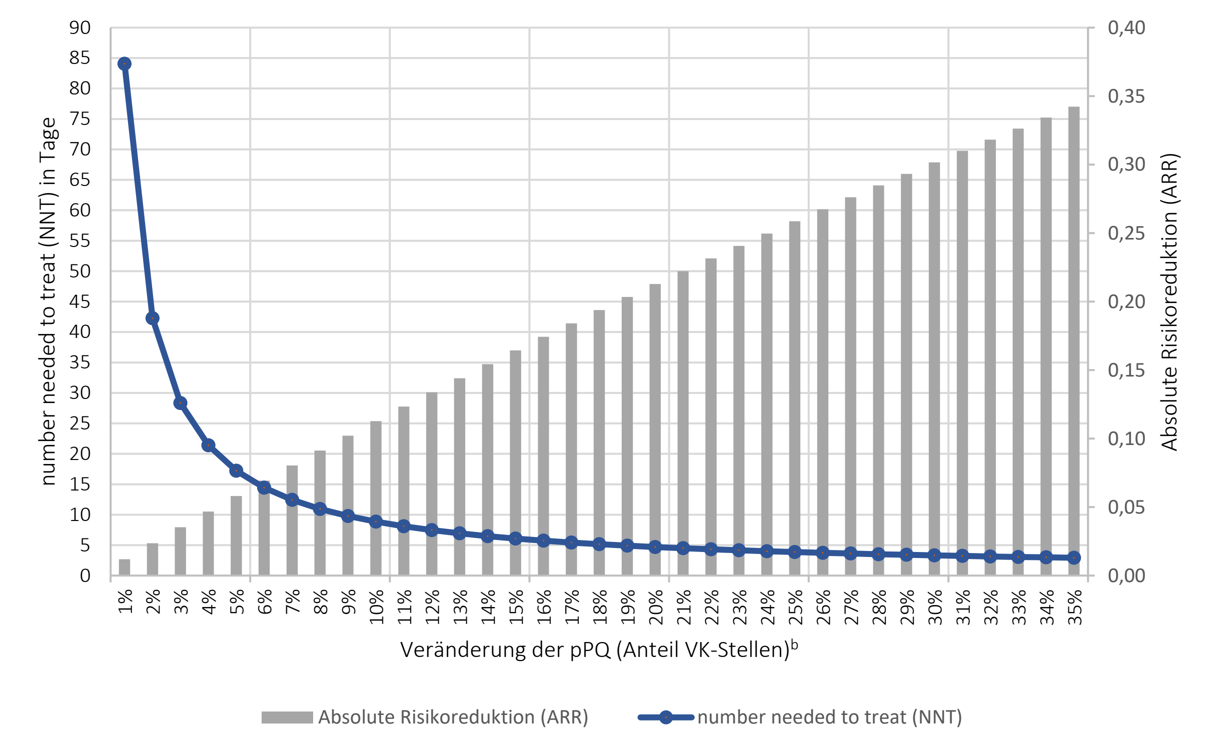

Anmerkungen: pPQ = Planerische Personalerfüllungsquote, VK-Stellen = Vollkosten-Pflegepersonalstellen

[a]Die Darstellung beruht auf den in *Zusatztabelle 2* und *Zusatztabelle 3* berechneten Regressionsmodellen.

[b]Die Veränderungen der pPQ bezieht sich auf den pPQ-Wert des Nullszenarios. Beträgt z. B. die durchschnittliche pPQ aktuell 80 % und es könnte eine Steigerung bzw. Veränderung um 10 % auf eine pPQ von 90 % erreicht werden, sinkt das Risiko für eine Neugeborenen-Intensivverlegung um 11,28 % (ARR). Dadurch kann im Sinne der NNT alle 8,9 Tage eine Intensivverlegung verhindert werden.

Die blau dargestellte Kurve zeigt den Verlauf der NNT-Werte bei Veränderung der pPQ gemäß der X-Achse. Die grauen Säulen verdeutlichen die absolute Reduktion des Risikos für eine Neugeborenen-Intensivverlegung bei Veränderung der pPQ gemäß der X-Achse. Es ist zu erkennen, wie effizient eine Erhöhung der pPQ beispielsweise um 10 % auf das Verlegungsrisiko wirkt, wobei das durchschnittliche Risiko für eine Verlegung um 11,3 % gesenkt werden kann, wodurch im Mittel alle 9 Tage eine Verlegung vermieden werden kann (NNT). Dagegen zeigen weitere Erhöhungen der pPQ im weiteren Verlauf eine deutlich niedrigere Wirkung.

Es ist allerdings in diesem Zusammenhang zu betonen, dass diese Interpretation allein auf den berechneten Modellen beruht und die im Artikel beschriebenen Limitationen dieser Studie zu beachten sind. Diese Darstellung verdeutlicht daher vor allem die Systematik dieser zweistufigen Regressionsanalyse.
